# The Evolutionarily Conserved LIM Homeodomain Protein LIM-4/LHX6 Specifies the Terminal Identity of a Cholinergic and Peptidergic *C*. *elegans* Sensory/Inter/Motor Neuron-Type

**DOI:** 10.1371/journal.pgen.1005480

**Published:** 2015-08-25

**Authors:** Jinmahn Kim, Jihye Yeon, Seong-Kyoon Choi, Yang Hoon Huh, Zi Fang, Seo Jin Park, Myoung Ok Kim, Zae Young Ryoo, Kyeongjin Kang, Hee-Seok Kweon, Won Bae Jeon, Chris Li, Kyuhyung Kim

**Affiliations:** 1 Department of Brain and Cognitive Sciences, Daegu Gyeongbuk Institute of Science and Technology (DGIST), Daegu, Korea; 2 Laboratory of Biochemistry and Cellular Engineering, Division of NanoBio Technology, Daegu Gyeongbuk Institute of Science and Technology (DGIST), Daegu, Korea; 3 Nano-Bio Electron Microscopy Research Group, Korea Basic Science Institute, Daejeon, Korea; 4 School of Life Sciences, KNU Creative BioResearch Group (BK21 plus program), School of Animal BT Science, College of Natural Sciences, Kyungpook National University, Daegu, Korea; 5 Department of Anatomy and Cell Biology, Samsung Biomedical Research Institute, Sungkyunkwan School of Medicine, Gyeonggi-Do, Korea; 6 Department of Biology, City College of the City University of New York, New York, New York, United States of America; University of California San Diego, UNITED STATES

## Abstract

The expression of specific transcription factors determines the differentiated features of postmitotic neurons. However, the mechanism by which specific molecules determine neuronal cell fate and the extent to which the functions of transcription factors are conserved in evolution are not fully understood. In *C*. *elegans*, the cholinergic and peptidergic SMB sensory/inter/motor neurons innervate muscle quadrants in the head and control the amplitude of sinusoidal movement. Here we show that the LIM homeobox protein LIM-4 determines neuronal characteristics of the SMB neurons. In *lim-4* mutant animals, expression of terminal differentiation genes, such as the cholinergic gene battery and the *flp-12* neuropeptide gene, is completely abolished and thus the function of the SMB neurons is compromised. LIM-4 activity promotes SMB identity by directly regulating the expression of the SMB marker genes via a distinct *cis*-regulatory motif. Two human LIM-4 orthologs, LHX6 and LHX8, functionally substitute for LIM-4 in *C*. *elegans*. Furthermore, *C*. *elegans* LIM-4 or human LHX6 can induce cholinergic and peptidergic characteristics in the human neuronal cell lines. Our results indicate that the evolutionarily conserved LIM-4/LHX6 homeodomain proteins function in generation of precise neuronal subtypes.

## Author Summary

The correct generation and maintenance of the nervous system is critical for the animal’s life. Dysregulation of these processes leads to multiple neurodevelopmental disorders. It has been a daunting challenge not only to identify the developmental mechanisms that determine neuronal cell fate, but also to understand the extent to which the mechanisms are evolutionarily conserved. Here, we describe a developmental mechanism that determines the fate of a specific cholinergic and peptidergic neuronal type in *C*. *elegans*. We show that the *lim-4* LIM homeodomain transcription factor is necessary and sufficient to promote and maintain the specific cholinergic and peptidergic properties and functions via binding to unique DNA sequences. We also demonstrate that *C*. *elegans lim-4* and human *LHX6* show striking functional similarity; specifically, *C*. *elegans* LIM-4 or human LHX6 can induce cholinergic and peptidergic characteristics in human neuronal cell lines. Given the high conservation of these transcription factors, these developmental mechanisms are likely to be generally applicable in the nervous system of other organisms as well.

## Introduction

The proper generation and maintenance of cells in the nervous system is essential for multi-cellular organisms. Each neuron achieves its identity by the acquisition of many distinct features, including appropriate synaptic contacts and expression of distinct sets of neurotransmitters. Fate determination and specification of neuronal cells largely relies on interactions between *trans*-acting transcription factors and *cis*-regulatory elements of their target genes [1, 2]. The same transcription factors may be used again after neuronal cell fate determination to maintain the neuron’s integrity [3]. However, it has been challenging not only to discover the transcription factors, but also to identify their regulatory mechanisms and target genes critically associated with determination and maintenance of neuronal cell fate.

In the nematode *Caenorhabditis elegans*, about 40% of nervous system (~120 neurons) appear to be cholinergic, including a subset of motor neurons in the ventral nerve cord and several sensory, motor and interneurons in the head, and many of these cholinergic neurons co-express neuropeptides [4, 5]. Several genes that function in terminal differentiation and specification of cholinergic neuronal fate have been identified, including the Olf/EBF transcription factor *unc-3* for the A-, B-, and AS-type ventral nerve cord and SAB motor neurons, LIM homeobox transcription factor *ttx-3* (ortholog of mammalian *Lhx2/9*) for the AIY and AIA interneurons, Paired-like homeobox gene *ceh-10* for the AIY interneurons, and POU homeobox gene *unc-86* for the IL2 sensory neurons, URA motor neurons and URB interneurons [6, 7, 8, 9, 10]. These transcription factors act as terminal selectors to directly or indirectly regulate expression of most terminal differentiation genes, such as the cholinergic gene battery but not that of pan-neuronal genes, and broadly affect terminal differentiation of each cholinergic neuron types [2]. Although the terminal selector transcription factors that are required for terminal differentiation of half of the cholinergic neurons have been identified, mechanisms and genes that differentiate other morphologically and functionally different cholinergic neuron types remain to be elucidated.

**Fig 1:**
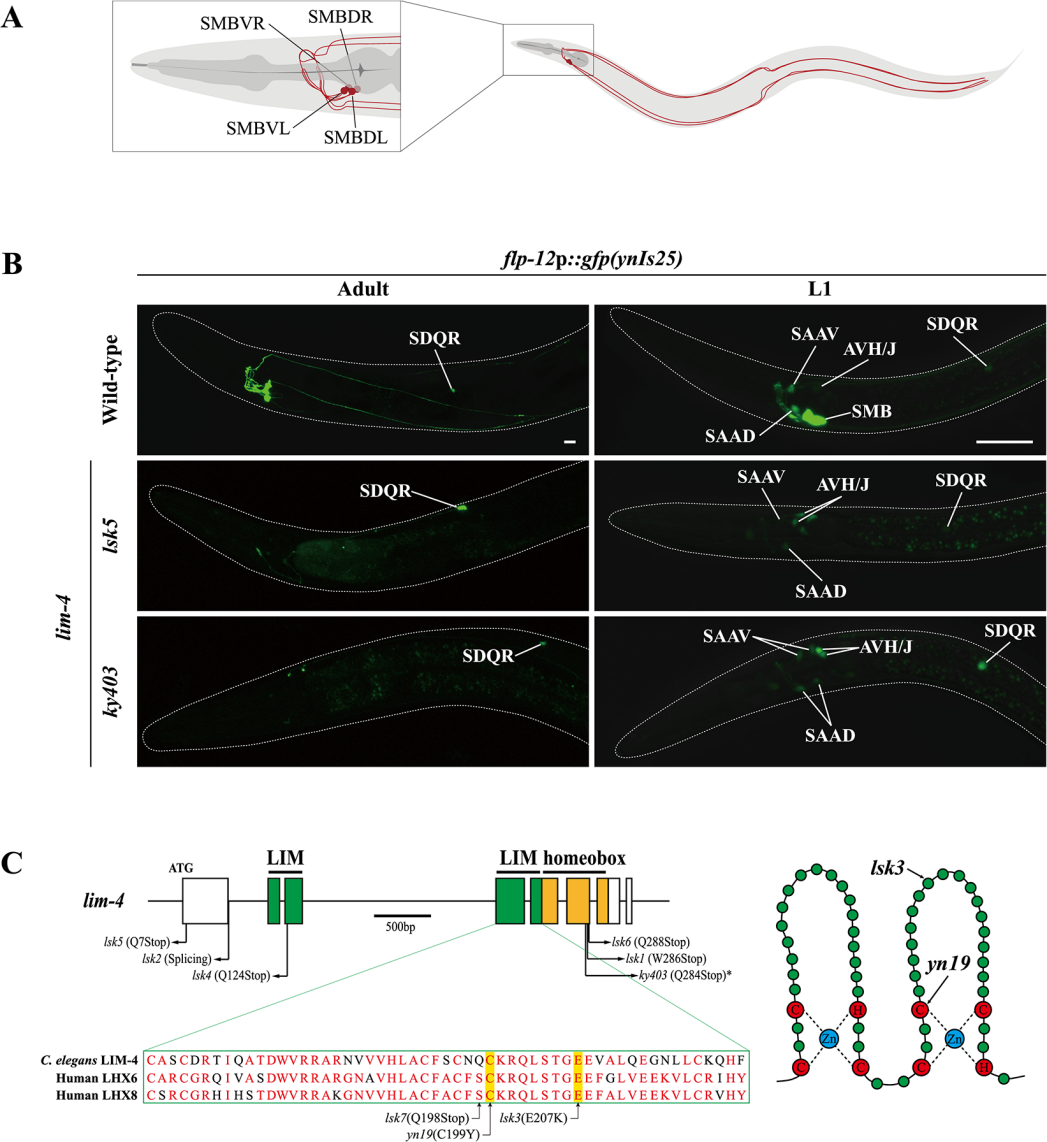
Expression of a *flp-12* neuropeptide reporter is abolished in the SMB neurons of *lim-4* mutants. (A) Schematic drawing of the SMB neurons in *C*. *elegans*. Four cell bodies are located in the head (DL: dorsal left, DR: dorsal right, VL: ventral left, VR: ventral right) and their processes run in sublateral cords to the tail. (B) Expression of the *flp-12* neuropeptide reporter is abolished specifically in the SMB neurons of *lim-4* mutants. GFP expression in *ynIs25* integrated strains is observed in the SMB and SDQ(L/R) neurons of wild-type adult (left column) or L1 larval (right column) stage animals while GFP expression in the SMB neurons is not detected in *lim-4* null mutant alleles (*lsk5*, *ky403*). Faint expression of the *flp-12*p::*gfp* reporter in the SAA and AVH/J neurons of L1 larvae is not altered in *lim-4* mutants. Anterior is at left in all images. Scale bars: 20 μm. (C) Genomic structure of *lim-4* (left) and schematic structure of the LIM domain of LIM-4 (right). The sequence alignment of part of the LIM domain of *C*. *elegans* LIM-4 and human LHX6 and LHX8 is shown. Identical residues in at least two proteins are shown in red. Molecular lesions of *lim-4* mutant alleles are indicated. *Mutation in *ky403* was previously reported [14]. LIM domain and homeodomain are labeled in green and in yellow, respectively.

The SMB multimodal sensory/inter/motor neurons consist of two pairs of neurons that are located in the head and innervate the head neck muscles (Fig 1A). Their processes, which run in ventral or dorsal sublateral cords to the tail and have electric and chemical synaptic contacts to other neurons in the head, were proposed to sense the stretch of body and regulate head locomotion [11]. In fact, laser ablation of the SMB neurons caused increased reversal frequency and wave amplitude of forward locomotion [12]. These neurons utilize at least two neurotransmitters, acetylcholine and a FMRFamide-related peptide, FLP-12 [5, 13]. Genes or molecules that are pivotal for the generation or differentiation of these SMB neurons have not been identified.

Here, we show that the LIM homeodomain LIM-4 protein is necessary to drive expression of terminal differentiation genes, including the cholinergic gene battery and the *flp-12* neuropeptide gene, but not pan-neuronal genes in the SMB neurons; consequently, in *lim-4* mutants, the neuronal function of the SMB neurons is abolished. We find that LIM-4 maintains its own expression by autoregulation in the SMB neurons and ectopic expression of LIM-4 is sufficient to drive expression of the SMB marker in other cell types. Moreover, our promoter analyses and bioinformatic searches with the SMB marker genes identified a *cis*-regulatory motif that is necessary and sufficient to drive gene expression in the SMB neurons. We also show that two *lim-4* human orthologs, *LHX6* and *LHX8*, functionally substitute for *lim-4* in *C*. *elegans*. Furthermore, expression of *C*. *elegans* LIM-4 or human LHX6 in the human neuroblastoma cell line induces cholinergic and peptidergic characteristics. We propose that there is an evolutionarily conserved role of *lim-4*/*LHX6*/*LHX8* LIM homeobox genes as terminal selectors to differentiate cholinergic and peptidergic neuronal cells and provide insight into how neuronal characteristics such as neurotransmitter identity are acquired via *trans*-acting and *cis*-regulatory mechanisms.

## Results

### Expression of a neuropeptide gene in the SMB neurons is abolished in *lim-4* mutants

To identify factors that specify the neuronal cell-fate of SMB, we performed a genetic screen to isolate animals in which the expression pattern of a terminal differentiation marker, *flp-12*p::*gfp* reporter, was disrupted exclusively in the SMB neurons. *flp-12* encodes a FMRFamide-related neuropeptide and is expressed in a set of neurons that includes the SMB and SDQ neurons in adults [13]. Among mutants isolated from this screen, seven mutant alleles (named as *lsk1*,*2*,*4*,*5*,*6*,*7*, *yn19*) exhibited complete loss of *flp-12* expression, while one mutant allele (*lsk3*) showed weak expression of *flp-12* in all four SMB neurons at either adult (Fig 1B; S1 Fig; Table 1) or L1 larval developmental stage (Fig 1B; Table 1; S1 Table) animals. By contrast, expression of *flp-12* in the SDQ and other neurons weakly expressing *flp-12* was unaffected in all eight mutants (Fig 1B; S1 Fig), indicating that expression of *flp-12* was specifically affected in the SMB neurons of these mutants.

**Table 1:** Expression pattern of the SMB expressed markers in *lim-4* mutant animals.

	Reporter construct	Gene product	Genotype	% animals showing GFP expression in SMB		
				Off	Weak	Strong
SMB-specific	*flp-12*p::*gfp*(*ynIs25*)	FLP-12 Neuropeptide	WT	0	0	100
			*ky403*	100	0	0
			*yn19*	100	0	0
			*lsk3*	77	20	3
			*lsk5*	100	0	0
			WT (L1)	0	0	100
			*ky403* (L1)	100	0	0
	*odr-2*p::*gfp*	Ly-6 superfamily	WT	0	0	100
			*ky403*	100	0	0
	*trp-1*p::*gfp*	TRP channel family	WT	0	100	0
			*ky403*	100	0	0
	*lim-4*p::*gfp*	LIM homeodomain protein	WT	0	0	100
			*lsk5*	100	0	0
			WT (L1)	0	0	100
			*lsk5* (L1)	0	6	92
Cholinergic	*unc-17*p::*gfp*	vesicular acetylcholine transporter	WT	0	0	100
			*ky403*	100	0	0
	*cho-1*p::*gfp*	choline transporter	WT	0	100	0
			*ky403*	93	7	0
Pan-neuronal	*rgef-1*p::*gfp*	Rap GRP	WT	0	0	100
			*ky403*	0	0	100
	*unc-119*p::*gfp*	chaperone	WT	0	80	20
			*ky403*	0	73	27

Expression pattern of GFP reporter constructs in the SMB neurons was observed in wild-type (WT) or *lim-4(ky403*, *yn19*, *lsk3*, *lsk5)* mutant animals at L1 larval stage (L1) or adult stage. Expression was observed at 630x or 400x magnification for L1 or young adults, respectively. Strong, weak or off expression is defined as GFP expression observed in both cell bodies and processes, observed in only cell bodies, or not observed either in cell bodies or processes, respectively. n≥30.

From subsequent complementation test and three factor analysis, all mutations were found to be allelic to the previously identified *lim-4(ky403)* mutation. *lim-4* encodes a LIM homeodomain protein that is required for specification of AWB and ADF chemosensory neuron identity [14, 15,16]. In *lim-4*(*ky403*) null mutants, AWB cell fate is changed to that of the AWC chemosensory neurons, thereby causing dye-filling defects in the AWB neurons [14]. Expression of *flp-12* was also completely abolished in the SMB neurons of *ky403* mutants (Fig 1B; Table 1). Like in the *ky403* mutants, the AWB neurons failed to dye-fill in the *lsk1-7* and *yn19* mutants (S2 Fig; S2 Table). The molecular lesions of all eight mutants mapped to the coding region of the *lim-4* gene (Fig 1C). Five mutant alleles (*lsk1*,*4*,*5*,*6*,*7*) had nonsense mutations that resulted in premature translation stop, suggesting that these mutations are null alleles. *lsk2* had a mutation in the splice donor site after the 1^st^ exon. *yn19* and *lsk3* had missense mutations within the coding region of the second LIM domain, resulting in C199Y and E207K substitutions, respectively. The cysteine residue (C199) is critical for forming a zinc finger motif in the LIM domain [15]. The glutamate residue (E207) resides in the LIM domain and is highly conserved through evolution (Fig 1C), suggesting that this residue is essential for LIM-4 function via protein-protein interactions. These findings indicate that LIM-4 has a role in regulating gene expression in the SMB neurons.

### Expression of terminally differentiated SMB markers including cholinergic genes is abolished in *lim-4* mutants

**Fig 2:**
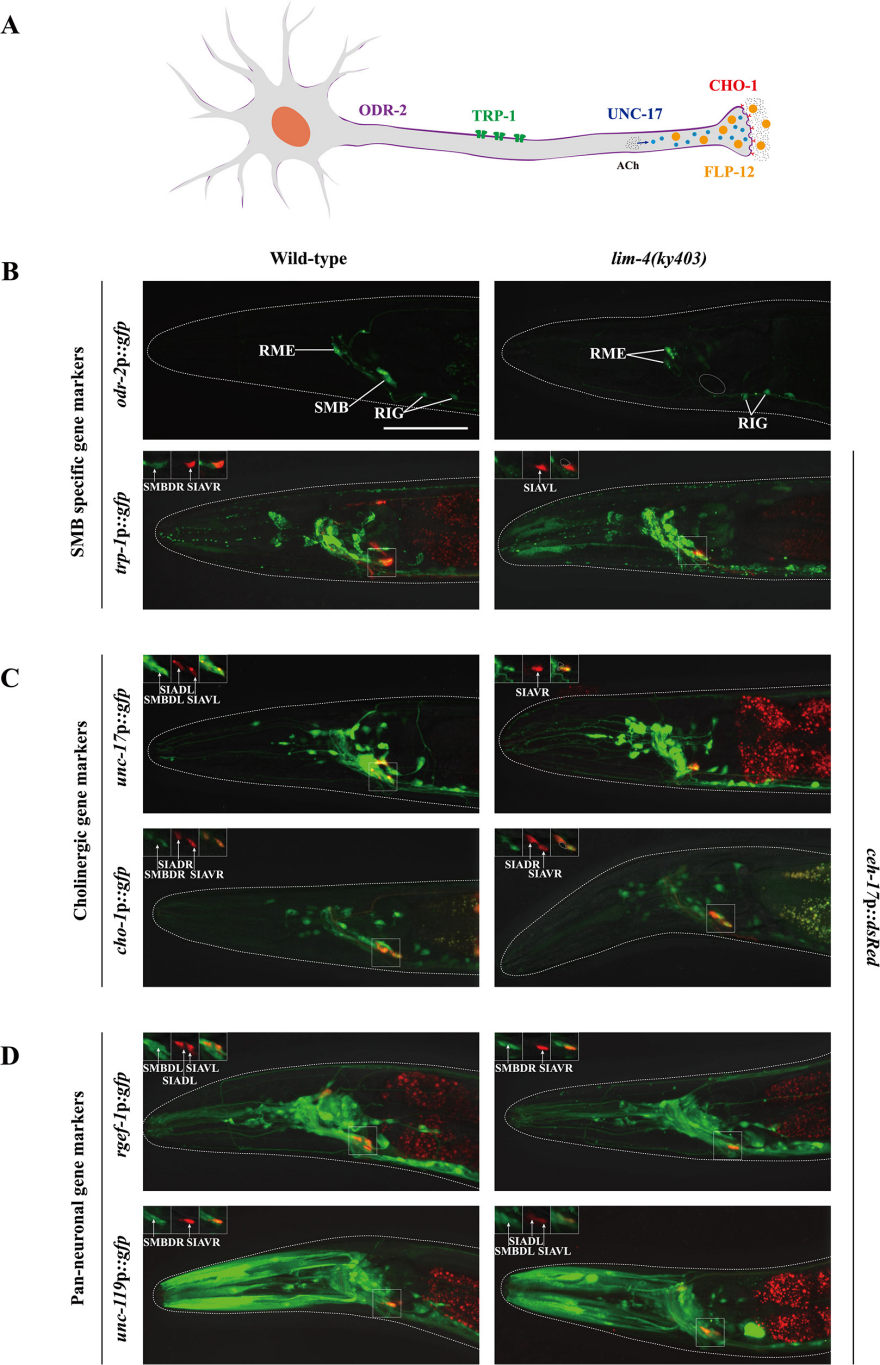
LIM-4 regulates expression of the terminally differentiated markers in the SMB neurons. (A) Schematic drawing of expressed genes in the SMB neurons; *odr-2* (GPI-anchored cell surface protein), *trp-1* (TRPC channel), *unc-17* (VAChT), *cho-1* (ChT), and *flp-12* (neuropeptide) (B-D) Expression of the indicated reporter constructs is shown in wild-type (left column) or *lim-4(ky403)* mutant (right column) animals. Merged images with the *ceh-17*p::*mCherry* reporter expression in the SIA neurons (shown in red) were shown for *trp-1*, *unc-17*, *cho-1*, *rgef-1*, or *unc-119* promoter reporter for help in identification of the SMB neurons (see S3 Fig). Images are derived from z-stacks of confocal microscopy images while images in the upper-left boxed regions are single focal plane confocal microscopy images. Quantitative analysis of these phenotypes is shown in Table 1. Anterior is to the left. Scale bar: 50 μm.

To determine the extent to which LIM-4 regulates gene expression in the SMB neurons, we examined additional SMB terminal differentiation genes, including *odr-2* GPI-anchored cell surface protein [17], *trp-1* TRPC channel [18], and cholinergic markers such as *unc-17* vesicular acetylcholine transporter (VAChT) [19] and *cho-1* choline transporter (ChT) (Fig 2A) [20]. In order to locate the SMB cell bodies, we used expression of *ceh-17*p::*dsRed* in the cell bodies of SIAV as a marker that is directly adjacent to the cell bodies of SMBD (S3 Fig) [21]. None of the SMB specific or cholinergic markers were expressed in the SMB neurons of *lim-4* mutants while expression in other neuron types was generally not affected (Fig 2B and 2C; Table 1). We next tested expression of two well-characterized pan-neuronal gene markers, *rgef-1* Ras guanine nucleotide releasing protein and *unc-119* chaperone [6, 7]. Expression of these pan-neuronal genes was not altered in the SMB neurons of *lim-4* mutants (Fig 2D; Table 1), indicating that the SMB cells may retain neuronal properties.

To determine whether the *lim-4* SMB neurons adopted a different cell fate such as the structurally and/or functionally related sub-lateral nerve cord neurons including the SIA, SIB, and SMD neurons, we tested markers including *ceh-17* for SIA [21], *flp-22* for SMD [13] or *ceh-24* for SIA, SIB, and SMD [22, 23]. None of these markers were ectopically expressed in *lim-4* mutants (S4 Fig), suggesting that the cell fate of the SMB neurons is not transformed to that of the structurally and/or functionally related cell types.

The SMB neurons are generated from ABalpapap (SMBDL, SMBVL) or ABarappap (SMBDR, SMBVR) precursors, and three of their sister cells undergo programmed cell death before hatching (S5 Fig) [24]. We observed expression of pan-neuronal markers in SMB of adult *lim-4* mutants (Fig 2D), suggesting that the SMB cells do not adopt the apoptotic fate of their sister cells. Based on these results, we conclude that LIM-4 activity does not initiate neuronal cell fate, but specifies SMB cell fate by regulating expression of terminal differentiation genes, thereby acting as a terminal selector transcription factor in the SMB neurons.

### The function of the SMB neurons is compromised in *lim-4* mutants

**Fig 3:**
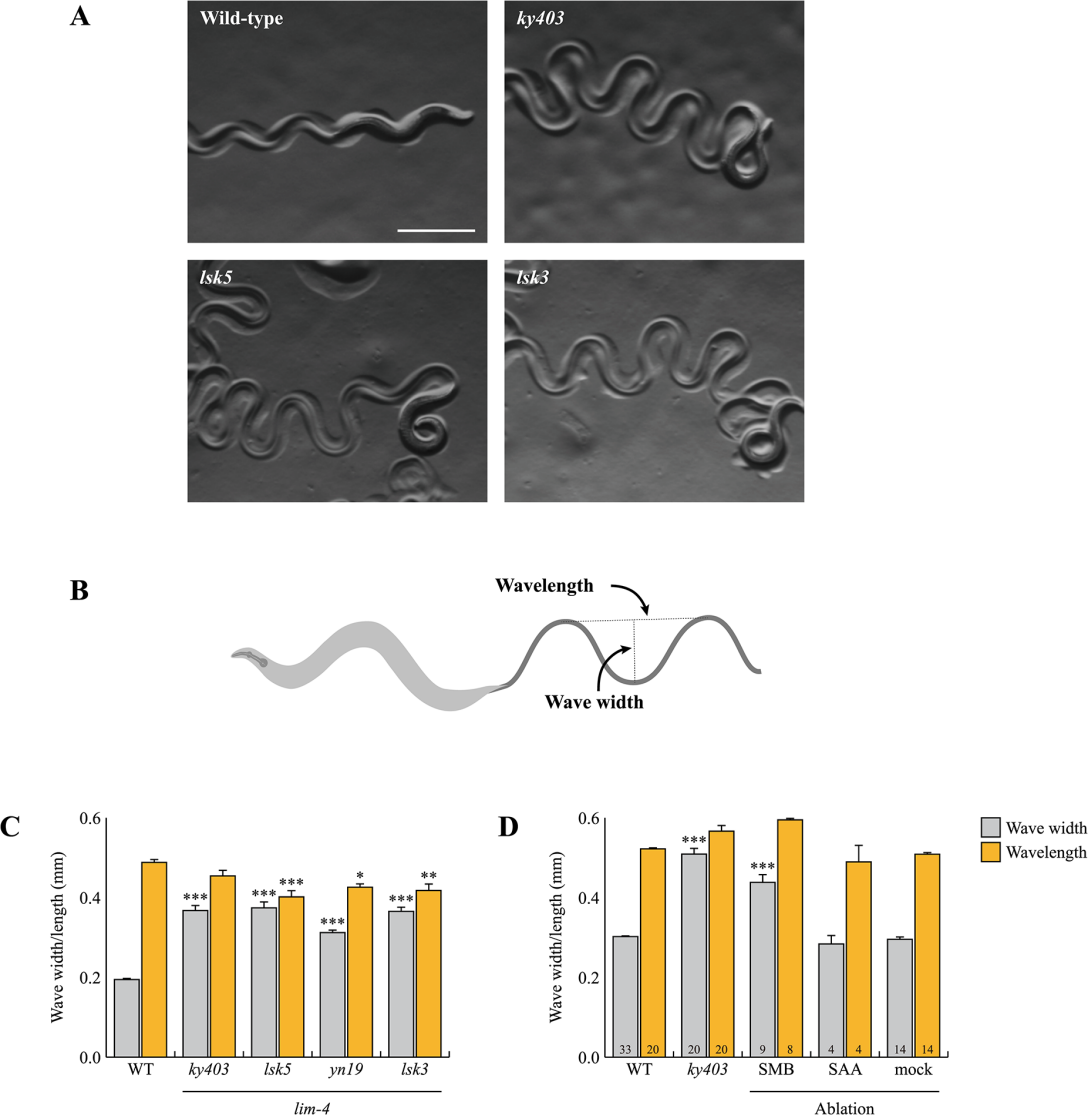
*lim-4* mutant animals moved in a coiled or loopy fashion due to the functional defects of the SMB neurons. (A) Wild-type animals show a characteristic sinusoidal waveform whose tracks can also be observed in the bacterial lawn. *lim-4*(*ky403*, *lsk5*, *lsk3*) mutant animals showed an exaggerated waveform characterized by an increased wave width. Scale bar: 0.5 mm. (B) To quantitate locomotion, the waveforms of different animals were analyzed and compared to that of wild-type animals by viewing tracks made in a bacterial lawn under a microscope. Wave width and wavelength were measured and averaged as the distance from the peak to the trough of the sine wave and distance between one peak and the next corresponding peak, respectively. (C-D) Average of wave width and wavelength of the *lim-4* mutant animals (C) or SMB/SAA-ablated animals (D). n≥30 for each. Error bars are the SEM. *, ** and *** indicate significantly different from wild-type at p<0.05, 0.01, and 0.001, respectively (one-way ANOVA test followed by the Tukey post-hoc test). The number of animals tested is indicated on the bars. Note that the size was measured by using the software (C) or a scale built into an eyepiece (D).

Wild-type animals move in sinusoidal waves of a consistent wave width and wavelength (Fig 3A) [25]. *lim-4* mutants move in a coiled or loopy fashion (Fig 3A) [14]. To quantitate the loopy uncoordinated movement, the waveforms of these animals were measured by viewing tracks made in a bacterial lawn and compared to that of wild-type animals (Fig 3B). *lim-4* mutants had significantly accentuated waveforms (Fig 3C). While the average wavelength for *lim-4* null mutants (*ky403* or *lsk5*) is similar or mildly decreased compared to that of wild-type animals, the average wave width for *ky403* or *lsk5* mutants (*ky403*: 359.46±13.51 μm, n = 30; *lsk5*: 374.27±16.47 μm, n = 30) is about 70% higher than that of wild-type animals (N2: 194.54±4.28 μm, n = 30) (Fig 3A and 3C). *yn19* and *lsk3* missense mutants similarly exhibited significantly larger wave width (*yn19*: 312.31±7.75 μm, n = 30; *lsk3*: 365.43±11.97 μm, n = 30) (Fig 3A and 3C), suggesting that *yn19* and *lsk3* mutations also fully eliminate the contribution of LIM-4 to locomotion.

To assess whether the loopy movement of *lim-4* mutants is due to a functional defect of the SMB neurons, we ablated the SMB neurons by laser microsurgery. Consistent with a previous study [12], killing the SMB neurons resulted in a loopy or coiled movement phenotype; the average wave width was increased by over 50% compared to control animals and similar to that of *lim-4* mutants (Fig 3D). We, however, noted that the SMB ablation did not result in as strong a loopy phenotype as the *lim-4* mutations, suggesting that the mutations have additional effects on locomotion beyond elimination of SMB function. *lim-4* is also expressed in the SAA neurons (see below) of which roles have been implicated in head locomotion [11]. Laser ablation of the SAA neurons did not cause a loopy or coiled movement, ruling out the possibility that defects of the SAA neurons result in movement defects of *lim-4* mutants (Fig 3D). These results indicate that the SMB neurons function to regulate locomotion by modulating the wave width of the animal and that the loopy phenotype of *lim-4* mutants is due to defects in the function of the SMB neurons.

### *lim-4* is expressed and functions in the SMB neurons to regulate their terminal specification

**Fig 4:**
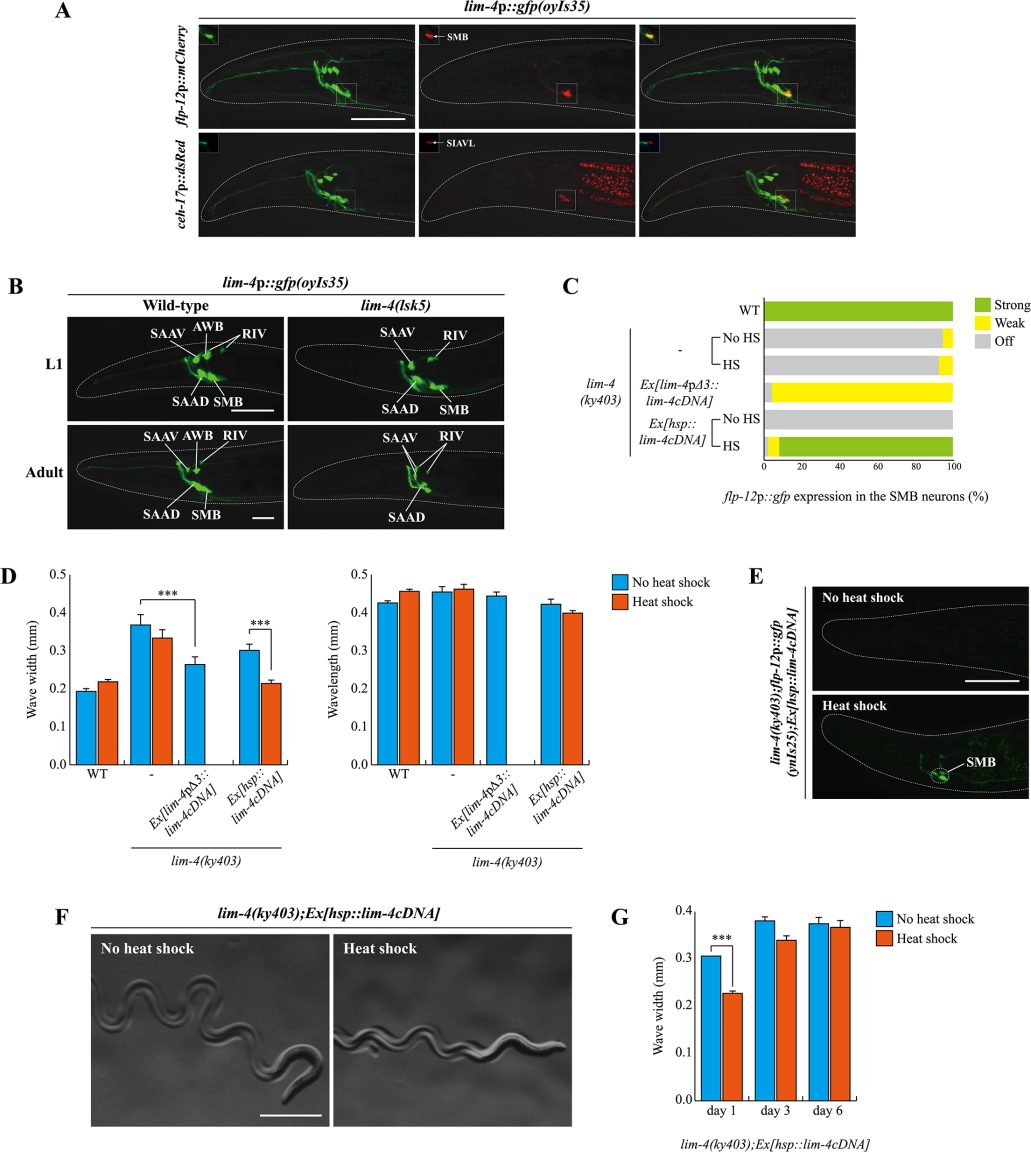
*lim-4* is expressed in and acts in the SMB neurons and its expression is autoregulated. (A) GFP expression of *oyIs35* animals carrying an integrated *lim-4*p::*gfp* reporter is overlapped with expression of the *flp-12*p::*mCherry* reporter (the SMB marker) but not with expression of the *ceh-17*p::*dsRed* reporter (the SIA marker). Images are derived from z-stacks of confocal microscopy images while images in the upper-left boxed regions are single focal plane confocal microscopy images. Anterior is to the left. Scale bar: 50 μm. (B) In *lim-4* mutants, expression of the *lim-4*p::*gfp* reporter is abolished in the cell bodies and processes of the SMB neurons in adult but not L1 larval stage animals. GFP expression of the *lim-4*p::*gfp* reporter is shown in wild-type (left column) or *lim-4*(*lsk5*) mutant (right column) animals at L1 larval (top) or adult stage (bottom). Note that the *lim-4*p::*gfp* reporter is not expressed in the AWB neurons of *lim-4* mutants [14]. Images are derived from z-stacks of confocal microscopy images. Quantitative analysis of these phenotypes is shown in Table 1. Anterior is to the left. Scale bar: 20 μm. (C) Percentage of animals of the indicated genotypes expressing stably integrated *flp-12*p:*gfp* reporter (*ynIs25*) is shown. Strong, weak or off expression is defined as GFP expression observed at 400x magnification in both cell bodies and processes, in only cell bodies, or not observed either in cell bodies or processes, respectively. Heat shocks were treated to L4 larval stage animals at 33°C twice for 30 minutes and after 14 hours, phenotypes were analyzed. Over two independent transgenic lines were tested. n≥50 for each. (D) The average of wave width or wavelength of the indicated genotypes. n≥30 for each. Error bars are the SEM. *** indicates significantly different between indicated animals at p<0.001 (one-way ANOVA test followed by the Tukey post-hoc test). Heat shocks were applied to L4 larval stage animals at 33°C twice for 30 minutes and phenotypes were analyzed after 14 hours. (E-F) Shown are representative pictures of *lim-4* mutants containing the *hsp*::*lim-4cDNA* transgene with no heat shock or after heat shock treatment. Images are derived from z-stacks of confocal microscopy images (E: Scale bar: 50 μm) and derived from a light microscopy image (F: Scale bar: 0.5 mm). (G) *lim-4* is required to maintain function of the SMB neurons. The average wave width was analyzed in *lim-4*(*ky403*);*Ex*[*hsp*::*lim-4cDNA*] at 1 day after, 3 day after or 6 day after heat shock treatment. n≥30 for each. Error bars are the SEM. *** Significantly different between no heat shock and heat shock treatment conditions at p<0.001(one-way ANOVA test followed by the Tukey post-hoc test).

*lim-4* has previously been shown to be expressed in several neuronal types in the head of postembryonic animals; these neurons include the AWB, SIA, SAA, RID, RIV, and RMD neurons, but not the SMB neurons [14]. Like the SMB neurons, the SIA neurons project their processes into the sub-lateral nerve cords and their cell morphology and position are similar to those of the SMB neurons [11]. To determine whether *lim-4* expression was mis-identified in the SIA neurons, we examined the expression pattern of *lim-4*p::*gfp* transgene (*oyIs35*) that includes 3.6 kb of upstream sequence [14, 26] and compared it to that of *ceh-17*p::*dsRed*, a SIA marker [21] or *flp-12*p::*mCherry*, a SMB marker [13], respectively (Fig 4A). We observed co-localization of *lim-4* expression with that of *flp-12* but not of *ceh-17*, indicating that *lim-4* is expressed in the SMB neurons rather than the SIA neurons. In support of this re-assignment, the expression of other SMB markers such as *odr-2*p::*gfp* or *trp-1*p::*gfp* was completely abolished in the SMB neurons of *lim-4* mutants, whereas expression of *ceh-17* was not affected (Fig 2B; S4 Fig).

Expression of *lim-4* was previously shown to be autoregulated in the AWB neurons but not in the other LIM-4-expressing neurons [14]. We confirmed that *lim-4* expression in the AWB neurons was not seen in *lim-4* mutants at L1 larval stage, whereas the expression of *lim-4* in the other neurons including the SMB neurons, was detected (Fig 4B). However, *lim-4* expression in the SMB neurons gradually decreased from the L1 larval stage until it became undetectable in the adult stage (Fig 4B; Table 1). Hence, *lim-4* appears to be required to maintain its own expression in the SMB neurons but does not initiate its expression, further supporting its role as a terminal selector gene in the SMB neurons.

To determine whether *lim-4* acts cell-autonomously within SMB, we tried to rescue *lim-4* phenotypes by expressing a wild-type *lim-4* cDNA driven under the control of *lim-4*p*Δ3* promoter. The *lim-4*p*Δ3* promoter includes minimal upstream regulatory sequences that drive transgene expression exclusively in the SMB neurons but not as strongly as the full promoter of *lim-4* and more dominantly in the SMBD than SMBV neurons (see below), and was used to identify expression in SMB of genes tested in this study (S6 Fig). The gene expression and locomotion defects of *lim-4* mutants were partially restored, while the dye-filling defects were still present and the normal average wavelength was not altered, indicating that LIM-4 acts in the SMB neurons to affect locomotion and transmitter specification (Fig 4C and 4D; S3 Table). Taken together, *lim-4* is expressed and acts in the SMB neurons to specify the SMB cell-fate.

### Postdevelopmental expression of LIM-4 is sufficient to restore the SMB-specific defects of *lim-4* mutants

To determine when the activity of LIM-4 is required for the expression of the SMB markers and proper locomotive movement, we first expressed *lim-4* with an inducible, ubiquitously expressed heat-shock promoter (*hsp16*.*2*) [27]. Upon transient supply of *lim-4* gene activity at the fourth larval stage (i.e., long after the SMB neurons have differentiated in the embryo), expression of *flp-12* was fully restored and the loopy phenotype of *lim-4* mutants was rescued (Fig 4C–4F). These results demonstrate that post-developmental expression of LIM-4 is sufficient to restore the expression of the SMB markers and the function of the SMB neurons in *lim-4* mutants. These data further indicate that the SMB neurons are not irreversibly switched to another cell-fate and demonstrate that loss of *lim-4* does not result in irreversible developmental defects. The dye-filling defects of the AWB/ADF neurons in *lim-4* mutants were partially rescued after multiple heat shocks (S3 Table) [14, 16].

We also used the inducible rescue assay to corroborate the prediction that *lim-4* is continuously required to maintain the functional properties of the SMB neurons. To this end, we supplied *lim-4* activity via the heat-shock promoter at L4 stage and then analyzed the animals after 14 hours at the young adult stage. In these animals, we found the locomotory defects to be partially rescued (Fig 4G). When assayed after a long time interval at 3 and 6 days (i.e., older adult stages), the animals again displayed a mutant phenotype indistinguishable from the control (Fig 4G), suggesting that the transient rescuing ability of the *lim-4* gene activity has faded. These results demonstrate that *lim-4* does not only initiate but also maintains the expression of the SMB terminal differentiation genes and, hence, the function of the SMB neurons.

### Expression of *lim-4* is sufficient to induce the SMB identity in other cell-types

**Fig 5:**
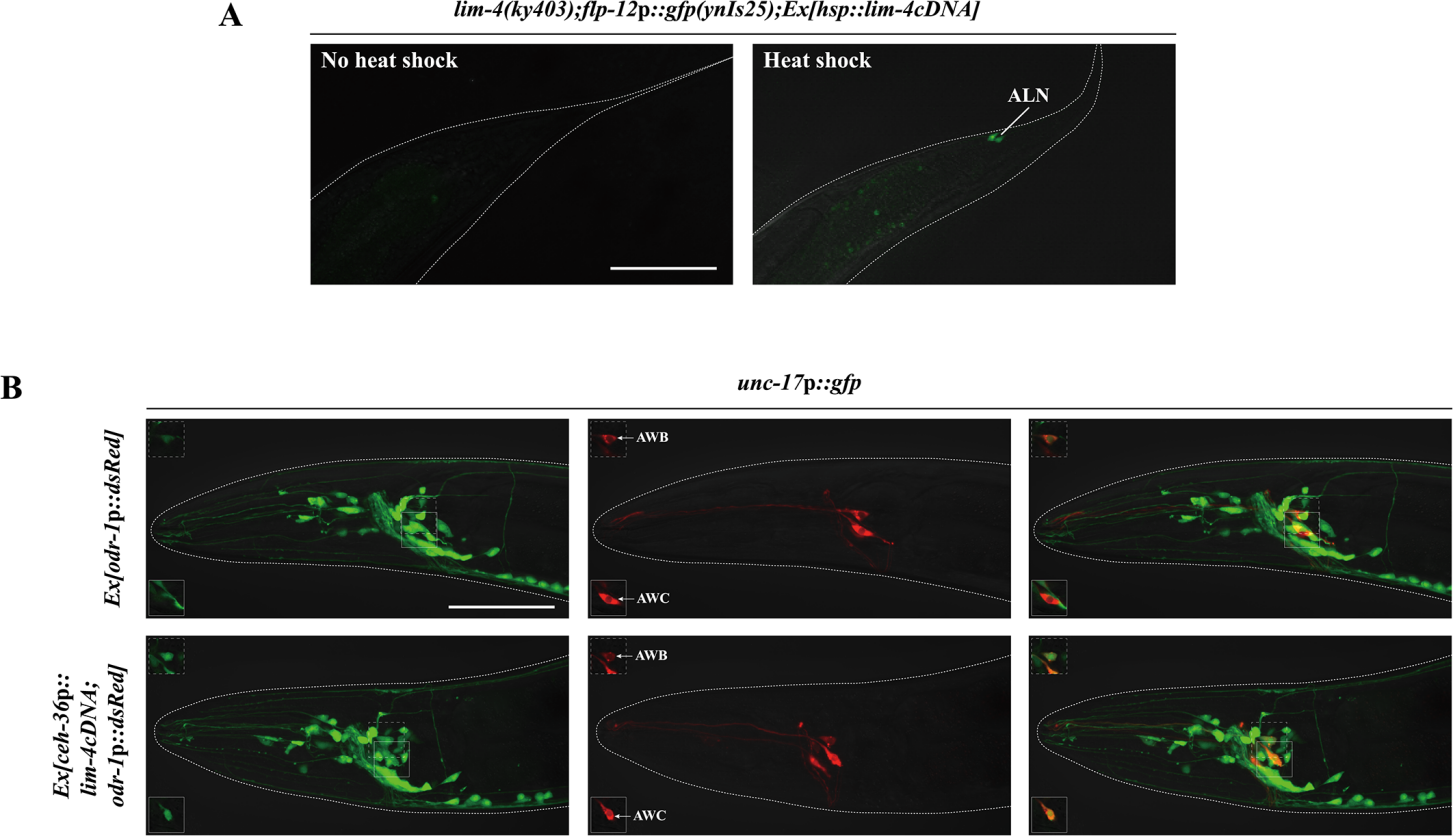
*lim-4* is sufficient to induce cholinergic or peptidergic marker expression in other cell-types. (A) Ectopic expression of LIM-4 is sufficient to drive *flp-12* expression in the ALN cholinergic neurons. Heat shocks were applied to 2 or 3 fold stage of embryos at the 37°C twice for 30 minutes and phenotypes of adults were analyzed. Images are derived from z-stacks of confocal microscopy images. Posterior is at right. Scale bar: 50 μm. (B) Ectopic expression of LIM-4 in AWC induces *unc-17* expression. *Ex[odr-1*p::*dsRed]* transgenic animals express dsRed in AWC and AWB [59]. Note that we observed *unc-17* expression in the AWB neurons of wild-type animals. Images are derived from z-stacks of confocal microscopy images while images in the upper or bottom-left boxed regions are single focal plane confocal microscopy images. Anterior is at left. Scale bar: 50 μm.

To address whether expression of *lim-4* is sufficient to induce the SMB identity in other cell-types, we first examined ectopic *flp-12* expression upon transient supply of *lim-4* gene activity via the heat-shock promoter at the embryonic stage. Although the heat-shock promoter should drive ubiquitous LIM-4 expression, ectopic *flp-12* expression was not seen broadly elsewhere; interestingly, expression was limited in only one cell-type, the ALN neurons, in 25% of transgenic animals (n = 50) (Fig 5A). The ALN neurons are a pair of cholinergic oxygen-sensing neurons in the tail [5, 11, 28] that do not appear functionally or linearly related to the SMB neurons (S5 Fig).

We next attempted to express LIM-4 in the glutamatergic chemosensory neurons AWC using the promoter of the *ceh-36* homeobox gene [26, 29]. Ectopic expression of *unc-17* VAChT was detected in AWC (Fig 5B) while the *flp-12* was not ectopically expressed in AWC (S7 Fig). We further induced broader ectopic expression of LIM-4 in the subset of glutamatergic neurons under a specific *eat-4* glutamate transporter gene promoter that drives reporter expression in 11 (but not in AWC) out of 38 glutamatergic neuron classes in the hermaphrodites [30]. Expression of LIM-4 in these cells did not drive ectopic expression of *cho-1* ChT or affect expression of *eat-4* (S8 Fig), suggesting that expression of LIM-4 alone is not sufficient to generally induce cholinergic cell fate in a subset of glutamatergic neurons. These results suggest that *lim-4* is partially sufficient to drive expression of the SMB markers in a context-dependent manner.

### LIM-4 regulates gene expression via a *cis*-regulatory motif in the SMB markers

**Fig 6:**
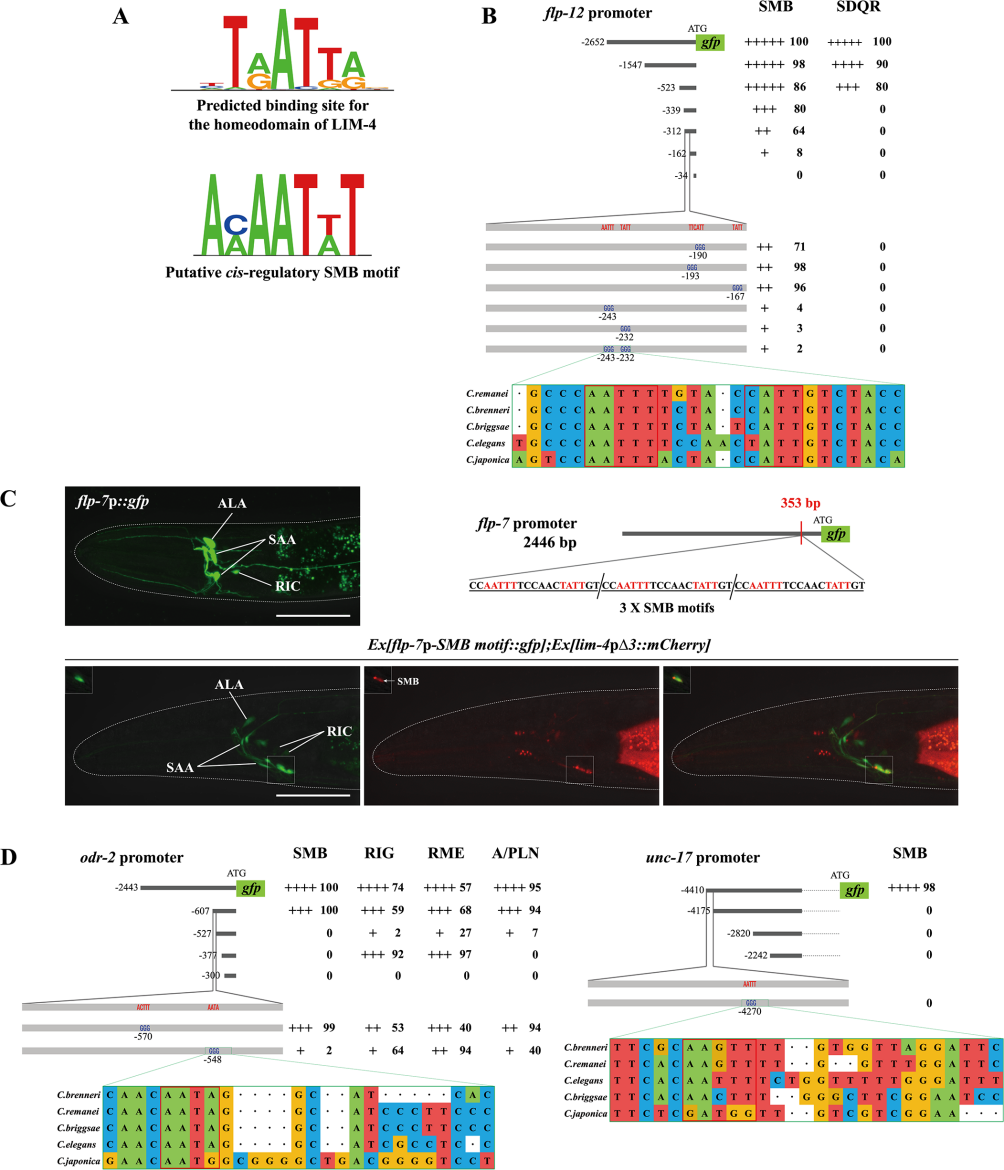
A *cis*-regulatory motif is necessary and sufficient to drive expression of terminally differentiated SMB markers. (A) Shown are the predicted binding site (top) for the homeodomain of LIM-4 from a web based tool, PreMoTF (http://stormo.wustl.edu/PreMoTF) [33] and the putative *cis*-regulatory SMB motif (bottom) identified from promoter analysis of *flp-12*, *odr-2*, and *unc-17* genes. (B) The percentage of transgenic animals expressing *flp-12*p:: *gfp* reporter construct in the indicated neurons is shown. Strength of GFP expression is indicated by the number of + symbols. Wild-type nucleotides are indicated in red, mutated nucleotides in blue. At least two independent extrachromosomal lines for each construct were examined except a *flp-12* promoter (-2652) construct of which number was derived from one integrated line. n≥50 for each. Identified *cis*-regulatory sequences found in other *Caenorhabditis* species are shown below. Analysis between -523 bp and -339 bp upstream of *flp-12* promoter is shown in S10 Fig. (C) Insertion of the SMB motif into the non-SMB expressed *flp-7* gene promoter induces *flp-7* expression in the SMB neurons. The inserted *cis*-regulatory sequences identified from the *flp-12* promoter analysis and the inserted site in the *flp-7* promoter are indicated. GFP expression is overlapped with expression of the *lim-4*p*Δ3*::*mCherry* reporter in the SMB neurons of wild-type animals. Images are derived from z-stacks of confocal microscopy images while images in the upper-left boxed regions are single focal plane confocal microscopy images. Anterior is to the left. Scale bars: 50 μm. (D) The percentage of transgenic animals expressing *odr-2*p::*gfp* or *unc-17*p::*gfp* reporter construct in the indicated neurons is shown. Strength of GFP expression is indicated by the number of + symbols. Wild-type nucleotides are indicated in red, mutated nucleotides in blue. At least two independent extrachromosomal lines for each construct were examined. n≥50 for each. Identified *cis*-regulatory sequences found in other *Caenorhabditis* species are shown below.

Homeodomain transcription factors generally bind well-defined DNA sequences to control transcription of target genes [31]. Systemic analysis of homeodomain DNA-binding specificities allowed prediction of the recognition motif of each homeodomain protein, and a *cis*-regulatory motif containing the consensus TAAT core DNA sequences was predicted to be the binding site for the homeodomain of LIM-4 and its mammalian and *Drosophila* homologs (LHX6/8 and Arrowhead, respectively) (Fig 6A; S9 Fig) [32, 33]. Indeed, LHX6 and LHX8 have been shown to directly bind to the predicted DNA sequences (ATAATCA) in the promoter regions of the *Shh* gene [34].

To identify *cis*-regulatory motifs required to drive expression of the SMB marker genes in the SMB neurons, DNA sequences within the promoters of the SMB markers were serially deleted and the resultant transgenic animals were examined for altered expression patterns. From these analyses, we first determined a minimal region within the *flp-12* promoter for *flp-12* expression. Deletion of a 150 bp sequence located ~162 bp upstream of the translation start sequence caused decreased *gfp* expression in the SMB neurons (Fig 6B; S10 Fig). Within the 150 bp region, we next found four AT- rich DNA sequences that are fully conserved in the promoters of the *flp-12* orthologs in the related *Caenorhabditis* species (Fig 6B). Mutations of two AT-rich DNA sequences resulted in an almost complete loss of *gfp* expression, while mutations of the other two sequences did not affect the *gfp* expression (Fig 6B), indicating that the former two motifs are necessary for the expression of *flp-12* in the SMB neurons. DNA sequences of these motifs (AAAATTG and ACAATAG) share limited sequence conservation with putative LIM-4 binding sequences, and will be referred to as SMB motifs (Fig 6A). To test whether these SMB motifs are sufficient to drive gene expression in the SMB neurons, we inserted three copies of the SMB motifs in the promoter of *flp-7*, which is normally expressed in the several head neurons, but not in the SMB neurons (Fig 6C) [13]. Transgenic animals expressing a *flp-7*p-SMB motif::*gfp* reporter construct still exhibited *gfp* expression in *flp-7* expressing neurons, indicating that insertion of the SMB motifs within the regulatory region of *flp-7* does not alter the *flp-7* expression pattern. In addition, we observed consistent expression of *flp-7* in the SMB neurons in 100% transgenic animals (n = 50) (Fig 6C), suggesting that these SMB motifs are necessary and sufficient to drive gene expression in the SMB neurons. These results are consistent with the hypothesis that LIM-4 directly binds the SMB motifs to regulate expression of *flp-12* gene in the SMB neurons.

To define additional regulatory motifs for expression of SMB markers, we examined the promoter regions of two additional SMB markers, *odr-2* and *unc-17*, and defined the regions essential for SMB expression. These regions in the *odr-2* or *unc-17* promoters contained the SMB motifs found in the *flp-12* promoter (Fig 6D). We mutated these motifs in the context of the *odr-2* and *unc-17* reporter genes and found that these mutations reduced expression of the reporter genes (Fig 6D). These results demonstrate that distinct *cis*-regulatory motifs can determine cell-specific expression or something of this sort.

A *cis*-regulatory region in the *lim-4* promoter that is required for *lim-4* expression in the AWB neurons was previously identified [35]. We performed analogous deletion analysis experiments in transgenic animals to dissect the *lim-4* promoter to identify motifs required for *lim-4* expression in the SMB neurons (S11 Fig). As proof-of-principle, we also identified the *lim-4* regulatory sequences for the AWB expression (S11 Fig). However, we could not identify simple *cis*-regulatory motifs in the *lim-4* promoter required for the SMB expression (S11 Fig). Instead, multiple regions in the *lim-4* promoter act in concert to regulate LIM-4 expression in the SMB neurons, suggesting a complexity of *cis*-regulatory motifs in the *lim-4* promoter to ensure proper LIM-4 expression in the SMB neurons.

### The function of *lim-4* is conserved in human

**Fig 7:**
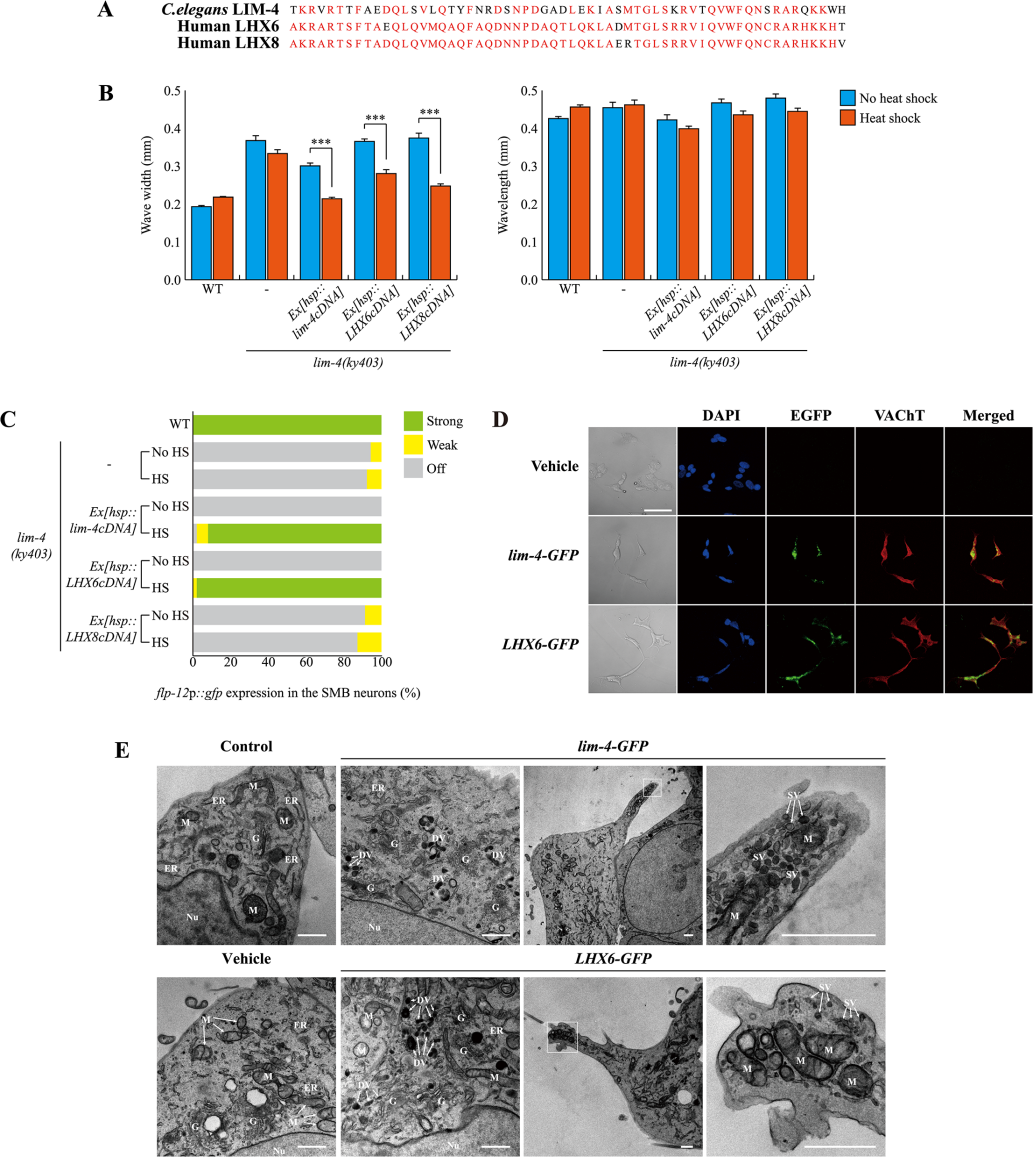
*C*. *elegans* LIM-4 or human LHX6 induces expression of cholinergic makers and neuronal characteristics in human neuroblastoma cells. (A) Shown is the sequence alignment of the homeodomain of *C*. *elegans* LIM-4 and human LHX6 and LHX8. Identical residues in at least two proteins are shown in red. (B) Average of wave width or wavelength of the indicated genotypes. n≥30 for each. Error bars are the SEM. *** indicates different between heat shock and no heat shock conditions at p<0.001 (student *t*-test). Heat shocks were applied to L4 larval stage animals at 33°C twice for 30 minutes and phenotypes were analyzed after 14 hours. Data of WT,-, or *Ex[hsp*::*lim-4cDNA]* are from Fig 4D. (C) Percentage of animals of the indicated genotypes expressing stably integrated *flp-12*p::*gfp* reporter (*ynIs25*) is shown. Strong, weak or off expression is defined as GFP expression observed at 400x magnification in both cell bodies and processes, in only cell bodies, or not observed either in cell bodies or processes, respectively. Over two independent transgenic lines were tested. n≥50 for each. Data of WT,-, or *Ex[hsp*::*lim-4cDNA]* are from Fig 4C. (D) Confocal images of SH-SY5Y human neuroblastoma cell line transfected by empty vector, *C*. *elegans lim-4* or human *LHX6* and immunostained with VAChT antibodies. Scale bar: 50 μm. Note that cells expressing either *LHX6-GFP* or *lim-4-GFP* exhibit spiky protrusions. (E) Ultrastructural analysis of LIM-4 or LHX6 transfected SH-SY5Y cell lines. Images from transmission electron microscope for untransfected (control), empty vector, *lim-4-GFP*, or *LHX6-GFP* transfected cells are shown. Left two columns are from peri-nuclear region and right two columns are from protruded region (far right images are higher magnification of the boxed area). Nu: nucleus, G: Golgi apparatus, M: mitochondria, ER: endoplasmic reticulum, DV: dense core vesicle, SV: synaptic vesicle. Scale bars: 1 μm. Confocal images of these control and transfected SH-SY5Y cells grown on the MatTek culture dish are shown in S14 Fig.

The mammalian genome contains two LIM-4 orthologs, *LHX6* and *LHX8*. In mice, these genes are largely expressed in the developing and adult striatum and orchestrate specification of interneuron identities; specifically, *LHX6* and *LHX8* are required to determine GABAergic/peptidergic and cholinergic interneuronal cell fate, respectively. In addition, these genes have redundant function to regulate expression of *shh* in MGE neurons [34]. LIM-4 exhibits a high degree of protein sequence homology to LHX6 and LHX8 (in particular, 60% identical in its homeodomain) (Fig 7A) [14], suggesting a functional conservation of these proteins.

To test for functional homology, we first tried to rescue *C*. *elegans lim-4* mutants by expressing human *LHX6 or LHX8* cDNA under the control of the heat shock promoter. Similar to *C*. *elegans lim-4* cDNA, human *LHX6 or LHX8* cDNA fully restored altered locomotion of *lim-4* mutants (Fig 7B; S12A Fig). Moreover, *LHX6* also fully rescued the defect of *flp-12* expression in *lim-4* mutants while *LHX8* did not rescue (Fig 7C; S12B Fig), indicating that LHX6 may have a higher degree of functional conservation to LIM-4 than LHX8.

LHX8 has been shown to be required for the development and maintenance of cholinergic neurons in mouse basal forebrain [36, 37]. Overexpression of LHX8 was sufficient to differentiate rat hippocampal neural stem cells or newborn neurons into cholinergic neuron types [38, 39] and induced expression of cholinergic markers in a human neuroblastoma cell line [40]. Whether LHX6 has a similar role in specification of cholinergic cell fate has not been explored. Thus, we tested whether overexpression of human LHX6 or *C*. *elegans* LIM-4 could promote expression of cholinergic markers in human neuroblastoma SH-SY5Ycells. These cells appear to mimic immature catecholaminergic neurons when untreated [41, 42], but can differentiate into various mature neuron-like phenotypes depending on the addition of differentiation-inducing agents [43]. We generated stable SH-SY5Y cell lines expressing either *LHX6-GFP*, *lim-4-GFP*, or empty vehicle and asked whether transfected cell lines express cholinergic markers. Expression of either *LHX6-GFP* or *lim-4-GFP* was detected predominantly in cell nuclei, supporting the action of LHX-6 and LIM-4 as transcription factors (Fig 7D; S13A Fig). Transfected cells were immunoreactive to VAChT or choline acetyltransferase (ChAT) antibodies and exhibited higher endogenous ChAT message levels, as assayed by quantitative reverse transcription polymerase chain reaction (RT-PCR), compared to that in cells transfected with an empty vehicle (Fig 7D; S13B Fig). Thus, human *LHX6* and even *C*. *elegans lim-4* are sufficient to promote expression of cholinergic markers in human cells. We also noted that cells expressing either *LHX6-GFP* or *lim-4-GFP* were morphologically different to cells bearing empty vehicle: empty vehicle bearing cells tended to grow in clusters and were round shape; by contrast, *LHX6-GFP* or *lim-4-GFP* expressing cells formed less clusters and appeared as spiky neuronal cells (Fig 7D; S13B Fig). These results, therefore, indicate that LHX6 or LIM-4 can induce differentiation of SH-SY5Y cells into cholinergic as well as neuronal phenotypes.

We further examined the morphology of transfected cells by electron microscopy. Untransfected or empty vesicle transfected SH-SY5Y cells exhibited typical shapes of mitochondria, endoplasmic reticulum (ER), and Golgi apparatus in the peri-nuclear region (Fig 7E). In either *lim-4* or *LHX6* transfected cells, however, we observed additional ultrastructural components, such as 100nm large-dense core vesicles, which may contain neuropeptides, near the Golgi apparatus (Fig 7E) [44]. Furthermore, in the spiky protruded region of *lim-4* or *LHX6* transfected cells, we also identified mitochondria and small synaptic vesicles that may represent axon terminals of neurons (Fig 7E). These results further support that expression of LIM-4 or LHX6 may produce synaptic vesicles containing large neuropeptides and small molecule neurotransmitters in human cell lines.

## Discussion

In these studies, we have identified an important regulator that controls terminal differentiation of a distinct neuronal cell-type in *C*. *elegans*. The SMB neurons appear to have mixed neuronal functions. Their long, unbranched and synapse-free processes along the body may serve as proprioreceptors to sense body stretch. In addition, they contact over 20 sensory, inter, or motor neurons via chemical or electrical synapses and may integrate additional extrinsic or intrinsic cues to regulate head muscle contraction [11]. The SMB neurons co-express a unique combination of neurotransmitters, acetylcholine and FLP-12 FMRFamide-like neuropeptides. Thus, an intriguing question is how the SMB sensory/inter/motor neurons acquire their unique characteristics. Our experiments show that the LIM-4 LIM homeodomain transcription factor is necessary and sufficient to promote and probably maintain SMB-specific properties and functions. In *lim-4* null mutants, the neurotransmitter identity and neuronal function of the SMB neurons are completely lost but pan-neuronal features are not affected. Transient LIM-4 expression in *lim-4* mutants not only restores the SMB characteristics and functions, but also induces ectopic expression of a SMB-expressed neurotransmitter in another cell-type. Our promoter analysis suggests that LIM-4 directly regulates expression of the SMB terminal differentiation marker via well conserved homeodomain binding sequences and also controls its own expression. Hence, we propose that *lim-4* acts as a terminal selector gene to broadly specify the SMB neuronal identity [2, 45].

A few terminal selector genes that determine cholinergic cell-fate in *C*. *elegans* have been identified. For example, the Olf/EBF gene *unc-3*, the heterodimer of LIM homeobox gene *ttx-3* and Paired-like homeobox gene *ceh-10*, and POU homeobox gene *unc-86* regulate terminal differentiation of the A-, B-, and AS-type ventral nerve cord or SAB motor neurons, the AIY interneurons, and the IL2 sensory neurons, URA motor neurons and URB interneurons, respectively [6, 7, 8, 9, 10]. *ttx-3* also acts as a terminal selector in the AIA interneurons [10]. These genes regulate expression of not only the cholinergic gene battery, including *unc-17* (VAChT), but also other terminally differentiated cell-specific markers. Furthermore, these *trans*-acting factors appear to directly bind to the evolutionarily conserved *cis*-regulatory elements of most, if not all, their target genes. In the case of the *unc-17* promoter region, distinct *cis*-regulatory target sites, such as the COE motif for UNC-3 and the AIY motif for TTX-3/CHE-10, are systemically organized (S15A Fig). In this study, we identified an additional *cis*-regulatory element, called the SMB motif, in the *unc-17* gene (S15A Fig), suggesting that the elaborate *cis*-regulatory architecture ensures expression of cell-specific characteristics. Since additional terminal selector genes required for specification of over 50 uncharacterized cholinergic cell-types need to be identified, additional motifs must exist in the cholinergic gene battery such as *unc-17* (S15A Fig).

Recent work has shown that distinct combination of 13 different terminal selector genes defines identity of 25 different glutamatergic cell-types in *C*. *elegans*, suggesting that the combinatorial codes of terminal selector transcription factors are a general theme for determining cell-type specificity [30]. In fact, in the AIY cholinergic neurons, two terminal selector transcription factors, *ttx-3* and *ceh-10*, form a heterodimer that directly regulates expression of their target genes via a common *cis*-regulatory bipartite motif; mutations of each gene lead to complete loss of the AIY specific neuronal identity [6, 9]. We have tried to identify the putative binding partner(s) of LIM-4 in the SMB neurons by analyzing the expression pattern of the *flp-12* reporter construct in mutants for which genes were previously reported to be expressed in the SMB neurons such as the *fax-1* nuclear receptor and *cog-1* Nkx6-type homeobox transcription factor [46, 47]. None of these mutations affects *flp-12* expression in the SMB neurons (S4 Table). Therefore, it is not yet clear which transcription factors work in combination with *lim-4* to control the terminal differentiation of the SMB neurons. Because our results demonstrate that *lim-4* expression is initiated at an early developmental stage and then autoregulated afterward, we also tested the possibility that expression of *fax-1* or *cog-1* in the SMB neurons may regulate *lim-4* expression in SMB. In chemosensory neuron types, the *lin-11* LIM homeobox, *ceh-37* Otx, *mls-2* HMX/NKX homeobox and *nhr-67* Tailless/TLX genes control expression of terminal selector genes in the AWA, AWB, AWC, and ASE neurons, respectively [26, 29, 48, 49]. However, *lim-4* expression is not altered in *fax-1* or *cog-1* mutants (S4 Table), indicating that these SMB-expressed transcription factors may have more specific roles in development or differentiation of the SMB neurons. We will continue searching for the transcription factors that partner with LIM-4 or act upstream or downstream of LIM-4 to control terminal differentiation of the SMB neurons.

Previous studies show that *lim-4* determines the proper cell-type specification of the AWB and serotonergic ADF neuron types [14, 16]. The AWB and ADF neurons are two classes of amphidial chemosensory neurons in the head of worms that detect volatile chemical repellants and putative food signals, respectively [50]. In *lim-4* mutants, the AWB neurons lack AWB-specific characteristics and functions, such as expression of putative 7-TM receptor *str-1*, AWB-specific cilia and axon morphology, and abilities to take up lipophilic dyes, and instead acquire features and functions of the AWC olfactory neurons, such as expression of the putative 7-TM receptor *str-2* and AWC-specific cilia and axon structures, suggesting that *lim-4* acts as a cell fate switch between the AWB and AWC neurons [14]. In case of ADF cell-fate specification, *lim-4* acts transiently in the precursor cells of ADF and regulates part of the terminal differentiation process; *lim-4* mutants lack expression of a set of serotonergic markers, including tryptophan hydroxylase *tph-1*, but do not affect expression of a putative 7-TM receptor *srh-142* [16]. The *ceh-37* Otx gene is required for expression of *lim-4* in AWB and *srh-142* in ADF, suggesting that *ceh-37* also differentially affects the AWB and ADF cell fates [26]. In addition, *lim-4* has a role in regulating axon morphology of the SAA neurons but not expression of terminal differentiation markers [14]. We propose that the SMB neurons in *lim-4* mutants do not adopt a functionally or lineage-related cell fate, but remained undifferentiated because they lose expression of most, if not all, terminal differentiation genes. Thus, *lim-4* plays distinct roles in neuronal development in a context-dependent manner and acts as a bona fide terminal selector for the differentiation of SMB (S15B Fig).

The LIM homeobox gene family has a high degree of structural conservation through evolution amongst the homeobox gene superfamily [51–53]. However, their functional conservation among distantly related species is relatively unexplored. We demonstrate that *C*. *elegans lim-4* and human *LHX6* and *LHX8 (*also referred as *L3* or *LHX7)* show striking functional similarity; LHX6 completely rescues locomotive defects and *flp-12* expression phenotypes of *lim-4* mutants, whereas human LHX8 restores only locomotion but not *flp-12* expression in *lim-4* mutants. Furthermore, expression of either *LHX6* or *lim-4* is sufficient to drive cholinergic differentiation in human neuroblastoma cells. The role of LHX8 in cholinergic cell-fate determination in the mammalian nervous system has been well characterized [36, 54, 55]. Deletion of the murine *LHX8* (*LHX7*), causes a subtype of cholinergic interneurons to convert into another subtype of GABAergic interneurons [55]. However, the study of LHX6 function has focused on GABAergic fate specification [56, 57]. We have uncovered that LHX6 also plays a role in cholinergic cell fate determination in *C*. *elegans* or human neuroblastoma cells and acts as a terminal selector to control the differentiation of neuronal subtypes. Cholinergic neurons in the mammalian forebrain have crucial roles in locomotive and cognitive functions and thus, understanding and manipulation of cholinergic cell fate specification may be beneficial to identify therapeutic targets and methods for neurodiseases resulting from cholinergic neuronal dysfunction.

## Materials and Methods

### Strains

N2 Bristol strain was used as wild-type strain. Mutant strains and transgenic strains used in this study are listed in S5 Table. All strains were maintained at 20°C.

### Isolation of *lim-4* mutants

The *flp-12*p::*gfp(ynIs25)* integrated strain was used to performed EMS mutagenesis according to Sulston and Hodgkin (1988). Eight alleles (*yn19*, *lsk1*, *lsk2*, *lsk3*, *lsk4*, *lsk5*, *lsk6*, *lsk7*) in which GFP expression was completely abolished, were isolated from screening ~15,000 haploid genomes, found to be allelic to each other, and mapped on LG X. Based on three factor crosses using the double mutants *unc-6 dpy-6*, *dpy-8 unc-6*, and *unc-2 dpy-8*, *yn19* was located approximately 2.4 MU downstream of *unc-2* and 4.3 MU upstream of *dpy-8*. In this region, we did complementation tests with a *lim-4(ky403*) mutant and found that they were allelic. The molecular lesions were identified by sequencing amplification products of *lim-4*. All *lim-4* alleles were outcrossed with N2 at least five times before phenotypic analysis. To observe locomotion phenotypes of *lim-4* mutants (*ky403*, *yn19*, *lsk3*, *lsk5*), the integrated *flp-12*p::*gfp* array was removed by mating with N2 males.

### Molecular biology and transgenic worms

For promoter analysis, promoter regions of *odr-2* and *unc-17* were amplified by PCR from N2 genomic DNA and were inserted into the pPD95.77 vector [27]. *lim-4*p::*gfp* [14] was gifted from Piali Sengupta. Promoter regions of each reporter construct were deleted with various digestion enzymes or PCR fusions. Mutagenesis was performed using QuikChange II XL Site-Directed Mutagenesis Kit (Stratagene) according to the manufacturer’s protocol.

For the constructs used to test for rescue, the *hsp16*.*2* promoter was fused with the following cDNAs: *lim-4* cDNA (kind gift form Oliver Hobert), human *LHX6* cDNA (BC103937) and human *LHX8* cDNA (BC040321) (Thermo Fisher Scientific). The *lim-4*p*∆3* or *odr-1* promoter were used to generate the l*im-4*p*∆3*::*lim-4cDNA* or *odr-1*p::*lim-4cDNA* constructs, respectively.

To generate the *flp-7*p-*SMBmotif*::*gfp* construct, three copies of SMB motif oligomers that have SacI enzyme site at 5’ and 3’ ends were synthesized and inserted into SacI site at -353 bp region of the *flp-7* promoter.

*otIs518; otIs534 (cho-1* ^*fosmid*^::*yfp; eat-4* ^*fosmid*^::*mChOpti)* and the *eat-4*p*∆5*::*gfp* construct were kind gifts from Oliver Hobert. To express *lim-4* cDNA in the subset of glutamatergic neurons, *lim-4* cDNA was replaced with *gfp* to generate *eat-4*p*∆5*::*lim-4cDNA*. Then, 2.5 ng of *eat-4*p*∆5*::*lim-4cDNA* was injected into the *otIs518; otIs534* strain with 50 ng *rol-6* as an injection marker.

To express *lim-4* cDNA in the AWC neurons during development stage, *ceh-36*p was fused with *lim-4* cDNA and 5 ng was injected into *vsIs48* (*unc-17*p::*gfp*) and *ynIs82 (flp-12*p::*gfp)* with 50ng of *odr-1*p::*dsRed* as an injection marker. As the control, 50 ng *odr-1*p::*dsRed* was injected into *vsIs48* (*unc-17*p::*gfp*).

*ceh-17*p::*dsRed* was kindly gifted from Satoshi Suo.

### Heat shock treatment and phenotype analysis

Heat shocks were administered to fourth larva stage (L4) of the transgenic worms at 33°C twice for 30 minutes with an hour incubation at 20°C between heat shocks for recovery modified from [7]. After heat shocks, worms were incubated at 20°C for 14 hours to reach the young adult stage when *lim-4* phenotypes were assayed. To observe ectopic expression of *flp-12*p::*gfp* in other cell types, heat shocks were administered at the embryo 2- or 3-fold stage two times at 37°C for 30 minutes with an hour incubation at 20°C between heat shocks. Heat shocks were administered at L4 animals to observe locomotion at days 1, 3, and 6. The L4 stage was counted as day 0.

The level of *flp-12*p::*gfp* expression in the SMB neurons was quantified as strong, weak, off. Strong was determined as robust expression in the SMB neuronal cell bodies and processes. Weak was defined as faint expression in cell bodies and no expression in the processes. Off was defined as no *flp-12*p::*gfp* expression in either cell bodies and processes. To measure wave width and wavelength of the worm tracks, Leica microscope software (Leica Application Suite Advanced Fluorescence Lite 3.5. Ink) was used. Wavelength was defined as distance between one peak and the next corresponding peak, and wave width was distance from the peak to the trough of the sine wave. The average of six consecutive wave width and wavelength from each worm track was quantified as the individual data.

### Laser ablation

Laser ablation experiments were performed as previously described [58]. L1 larvae of the integrated *lim-4*p::*gfp* (*oyIs35*) strain were anesthetized with 10 mM sodium azide and all four SMB or SAA neurons were killed by a nitrogen dye-pulsed laser (Photonic Instruments, St. Charles, IL). Animals were recovered for 3 days at 20°C. After performing locomotion assays, GFP expression of *lim-4*p::*gfp* reporter was observed to confirm laser ablations in the SMB or SAA neurons.

### Bioinformatics analysis

The conservation of the *cis*-regulatory motif from the promoter analysis of the *flp-12*, *lim-4*, *odr-2*, and *unc-17* were examined by using the USCS genome browser (http://genome.ucsc.edu/). DNA sequences from the five different *Caenorhabditis* species were obtained from the UCSC website, and aligned using the ClustalW2 in EBI (European Bioinformatics Institutes; http://www.ebi.ac.uk/Tools/msa/clustalw2/) to identify *cis*-regulatory regions including SMB motifs. The position frequency matrix (PFM) of LIM-4, LHX6, and LHX8 predicted binding sites were derived from a web based tool, PreMoTF (http://stormo.wustl.edu/PreMoTF). Predicted conserved motif sequence logo was obtained from the Seq2Logo website (http://www.cbs.dk/biotools/Seq2Logo/).

### Microscopy

Fluorescent microscopic images were taken with a Zeiss LSM700 Confocal microscope and were obtained using ZEN 2009 Light Edition software. For light microscopic images of worms, Leica High-performance Fluorescence Stereomicroscopy M205FA was used and Leica Application Suite Advanced Fluorescence Lite 3.5 software was used to measure the phenotype.

### Cell culture

The SH-SY5Y human neuroblastoma cell line (ATCC) was cultured with 10% complete medium (1:1 mixture of DMEM and Ham's F12 medium and 10% supplemental fetal bovine serum, 100 U/ml penicillin, and 100 μg/mL streptomycin) in a humidified, 5% CO2-95% air, 37°C incubator.

### Construction recombinant lentiviruses and transfection into the SH-SY5Y cells

HEK293T cells were purchased from American Type culture Collection (ATCC) and were cultured in DMEM with 10% FBS. cDNA for *lim-4* or *LHX6* was cloned into the lentiviral vector, pRetroX-IRES-ZsGreen1 (Clontech). Transformation was performed with lentivirus constructs and packaging vector into 293T cells using a lipofectamine 2000 reagent (Invitrogen). At 72 hours post transfection, the viral particles were harvested by filtration using a 0.45mm syringe filter.

Wells of glass bottom dishes (MatTek) were coated 0.1μM fibronectin (Sigma) for 1 h at 37°C. After removing the fibronectin solution, SH-SY5Y cells were seeded at density of 5×10^4^ cells per well and grown to confluence in 50%. Viruses were added to cells at 37°C for 120 min followed by addition of an equal volume of DMEM and Ham's F12 medium with 10% fetal bovine serum, and incubation for a further 24 h. Cells then were washed with phosphate-buffered saline, and DMEM and Ham's F12 medium with 1% fetal bovine serum. After an additional 24 h in growth medium, cells were washed in 1:1 DMEM and Ham's F12 medium with 1% fetal bovine serum. Under these conditions, up to 90% of cells were infected.

### Immunocytochemistry

Cells were fixed using 4% paraformaldehyde, followed by 0.1% triton-X permeabilization and incubation with antibodies. Fixed cells were incubated at 4°C overnight with the primary antibodies, including human-cross reactive rabbit anti-ChAT (Millipore) and human-cross reactive rabbit anti-VAChT (Synaptic Systems). Bound antibodies were visualized with Alexa Fluor 594 FluoroNanogold-anti-rabbit–conjugated secondary antibodies (Nanoprobes). Nuclei were stained with 4′,6-diamidino-2-phenylindole (DAPI, Sigma).

### qRT-PCR

Total RNA was obtained from the cells by using a Trizol Reagent (Invitrogen) according to the manufacturer’s instructions. cDNAs were synthesized using High-Capacity cDNA reverse transcription kits (Applied Biosystems). Quantitative real-time RT PCR was performed using the SYBR Green PCR master mix kit (Applied Biosystems) on the ABI 7500 Real Time PCR System under the following conditions: Cycling conditions were 2 min at 50°C, 10 min at 95°C, followed by 40 cycles of 95°C for 15s and 60°C for 1 min. Primer sets were designed using primer express 3.0 software based on the human gene sequences from GenBank and are as follows: ChAT (sense: 5'-GGCTCAGAACAGCAGCATCA -3' and antisense: 5'-GAGACGGCGGAAATTAATGACA -3'); GAPDH (sense: 5'-ACCCACTCCTCCACCTTT GA-3' and antisense: 5'-TGTTGCTGTAGCCAAATTCGTT-3'). The housekeeping gene GAPDH was used as an internal standard. Reaction specificity was confirmed by melting curve analysis.

### EM

Control and transfected SH-SY5Y cells grown on the MatTek culture dish were fixed for 2 h at 4°C in PBS containing 2.5% glutaraldehyde. After three washes in PBS, the cells were postfixed with 1% osmium tetroxide on ice for 2 h and washed three times again in PBS. The cells were then embedded in Epon 812 mixture and polymerized in an oven at 60°C for 24 hours after dehydration in increasing concentrations of ethanol (50, 70, 80, 90, 95 and 100%) and propylene oxide series (20 min each). The embedded blocks were trimmed and sectioned on an ultramicrotome with a diamond knife and ultrathin sections were collected on Formvar-coated copper grids. The grids were stained with 2.5% uranyl acetate (7 min) and Reynolds lead citrate (2 min), and were viewed with a transmission electron microscope (Technai G2 Spirit Twin, FEI, USA) at 120 kV.

## Supporting Information

S1 Table Expression pattern of the *flp-12* in *lim-4* mutant animals at L1 stage. Expression pattern of *flp-12*p::*gfp* reporter constructs in the SMB neurons was observed in wild-type (WT) or *lim-4* (*lsk3*, *lsk5*, *yn19*) mutant animals at L1 larval stage (L1). Expression was observed at 630x. Strong, weak or no expression is defined as GFP expression observed in both cell bodies and processes, observed in only cell bodies, or not observed either in cell bodies or processes, respectively. n≥50.
(PDF)

S2 Table Dye-filling defects in the AWB neurons of *lim-4* mutant animals. Lipophilic dye DiD was used to observe dye-filling in the AWB neurons of *lim-4* mutant animals (*ky403*, *yn19*, *lsk3*, *lsk5*). In *lim-4* (*ky403*, *lsk5)* null mutants, the AWB neurons are not dye-filled, while the ADF neurons are ectopically dye-filled [16]. Dye staining in adult animals was observed at 400x. n≥30.
(PDF)

S3 Table Dye-filling defects of *lim-4* mutants are partially rescued by temporal expression of LIM-4. Lipophilic dye DiD was used to observe dye-filling in the AWB/ADF neurons of *lim-4* mutant animals. Dye staining in adult animals was observed at 400x. n≥30.
(PDF)

S4 Table The expression of the *flp-12* or *lim-4* in the SMB neurons is not altered in *fax-1* or *cog-1* mutant animals. Expression pattern of *flp-12*p::*gfp or lim-4*p::*gfp* reporter constructs in the SMB neurons was observed in wild-type (WT), *fax-1* (*gm83*) or *cog-1*(*sy275*) mutant animals. Expression was observed at 400x. Strong, weak or no expression is defined as GFP expression observed in both cell bodies and processes, observed in only cell bodies, or not observed either in cell bodies or processes, respectively. n≥50.
(PDF)

S5 Table Transgenes and strains used in this study. (PDF)

S1 Fig Expression of a *flp-12*p::*gfp* reporter is abolished in adult *lim-4* mutants. Two integrated strains expressing a *flp-12*p::*gfp* reporter (*ynIs82* or *ynIs25*) were used for EMS mutagenesis screens. Note that *ynIs82* integrated strains exhibit variable and weak GFP expression in a few neurons of the head. Anterior is at left in all images. Scale bars: 50 μm.
(PDF)

S2 Fig The AWB neurons fail to be dye-filled in *lim-4* mutants. The AWB neurons and additional five pairs of amphid neurons (ASK, ADL, ASI, ASH, ASJ) in the head of wild-type animals fill with lipophilic dye DiD [60]. In *ky403* and eight newly identified *lim-4* alleles, the AWB neurons fail to be dye-filled. Images are derived from z-stacks of confocal microscopy images taken for left-side amphid neurons. Dashed circles indicate position of the AWB neurons. Anterior is at left in all images. Quantitative analysis of these phenotypes is shown in S2 Table. Scale bar: 50 μm.
(PDF)

S3 Fig Identification of the SMB cell bodies. Shown are the images of expression of *flp-12*p::*gfp* (SMB) or *ceh-17*p::*dsRed* (SIA), the merged images, or schematic drawing of cell bodies in the merged images. Top images are from lateral view and bottom images are from ventral view of head of worms. Although positions of the cell bodies of SMBs or SIAs are variable, we consistently observed that the cell bodies of SMBDL/R and SIAVL/R are located immediately adjacent to each other at the same focal plane in all tested animals (n>30). Therefore, we identified SMBDL/R cell bodies via Nomarski optics by comparing with expression of *ceh-17*p::*dsRed* in the cell body of SIAVL/R. Images are derived from z-stacks of confocal microscopy images while images in the upper-left boxed regions are single focal plane confocal microscopy images. Anterior is at left in all images. Scale bar: 50 μm.
(PDF)

S4 Fig The SMB neurons in *lim-4* mutants do not adopt the cell-fate of the SIA, SIB, or SMD neurons. Expression of the indicated reporter constructs is shown in wild-type (left column) or *lim-4(ky403)* mutant (right column) animals. Expression of the *ceh-24*p::*gfp* reporter is detected in about 8 cells bodies in the head of worms including the SIA, SIB, and SMD neurons but not the SMB neurons. Images are derived from z-stacks of confocal microscopy images. Anterior is to the left. Scale bar: 50 μm.
(PDF)

S5 Fig The cell lineage of the SMB neurons and ALN neurons in wild-type animals. Precursor cells of the SMB neurons are shown in yellow circles. x indicates the programmed cell death. Heat shocks to transgenic animals expressing *hsp*::*lim-4 cDNA* transgene induced *flp-12* expression in the ALN neurons (see Fig 5A).
(PDF)

S6 Fig The SMB neurons express cholinergic or pan-neuronal markers. Expression of *gfp* reporter constructs under the control of cholinergic (*unc-17*, *cho-1*), SMB-specific (*trp-1*, *odr-2*) or pan-neuronal (*rgef-1*, *unc-119*) gene promoter is overlapped with expression of the *lim-4*p*Δ3*::*mCherry* reporter in the SMB neurons of wild-type animals. Expression of *acr-2*, *acr-5*, *acr-14*(AChRs), or *ace-2* (AChE) cholinergic marker is not detected in the SMB neurons. Expression of the indicated reporter constructs and of the *lim-4*p*Δ3*::*mCherry* reporter is shown in left or middle column, respectively, and the merged images are shown in right column. Images are derived from z-stacks of confocal microscopy images while images in the upper-left boxed regions are single focal plane confocal microscopy images. Anterior is at left in all images. Scale bar: 50 μm.
(PDF)

S7 Fig Ectopic expression of LIM-4 in the AWC neurons does not drive *flp-12* expression in the AWC neurons. *ceh-36* gene promoter drives LIM-4 expression in the AWC and ASE neurons [26]. *Ex[odr-1*p::*dsRed]* transgenic animals express dsRed in AWC and AWB [59]. Anterior is at left in all images. Scale bar: 50 μm.
(PDF)

S8 Fig Ectopic expression of LIM-4 does not drive *cho-1* expression in the subset of glutamatergic neurons. *eat-4*p*Δ5* promoter drives LIM-4 expression in 11 out of 38 glutamatergic neuron types [30]. Images are derived from z-stacks of confocal microscopy images. Anterior is at left in all images. Scale bar: 50 μm.
(PDF)

S9 Fig The predicted binding sites for the homeodomain of Arrowhead, LHX6, or LHX8. The binding sites are derived from a web based tool, PreMoTF (http://stormo.wustl.edu/PreMoTF) [33].
(PDF)

S10 Fig Analysis between -523 bp and -339 bp upstream of *flp-12* promoter. A *cis*-regulatory sequence for the SMB neurons was not identified in this promoter region. The percentage of transgenic animals expressing each *gfp* reporter construct in the indicated neurons is shown. Strength of GFP expression is indicated by the number of + symbols. Point mutated nucleotides are indicated as wild-type in red line. At least two independent extrachromosomal lines for each construct were examined. n≥50 for each.
(PDF)

S11 Fig Analysis of the *lim-4* gene promoter. The percentage of transgenic animals expressing each *gfp* reporter construct in the indicated neurons is shown. Strength of GFP expression is indicated by the number of + symbols. Deleted regions in the promoter are indicated as a dotted line. Mutated nucleotides are indicated as wild-type in red line. The *cis*-regulatory sequence for the AWB expression of *lim-4* is indicated. At least two independent extrachromosomal lines for each construct were examined. n ≥50 for each.
(PDF)

S12 Fig Representative pictures of *lim-4* mutants containing the *hsp*::*LHX6cDNA* or *hsp*::*LHX8cDNA* transgene with no heat shock (left) or after heat shock (right) treated conditions. Images are derived from a light microscopy image (A: Scale bar: 0.5 mm) and from z-stacks of confocal microscopy images (B: Scale bar: 50 μm).
(PDF)

S13 Fig *C*. *elegans* LIM-4 or human LHX6 induces expression of ChAT and neuronal characteristics in human neuroblastoma cells. (A) Confocal images of SH-SY5Y human neuroblastoma cell line transfected by *C*. *elegans lim-4* or human *LHX6* and immunostained with ChAT antibodies. Scale bar: 50 μm. (B) Levels of ChAT transcripts are increased in SH-SY5Y human neuroblastoma cell line transfected by *C*. *elegans lim-4* or human *LHX6*. The relative fold change to housekeeping gene GAPDH is shown. *** indicates significantly different from control (untransfected cells) (p<0.001).
(PDF)

S14 Fig Confocal images of untransfected (control) and vehicle, *lim-4* or *LHX6* transfected SH-SY5Y cells grown on the MatTek culture dish are shown. Scale bar: 500 μm
(PDF)

S15 Fig Model for the *cis*-regulatory architecture underlying cholinergic cell fate specification in *C*. *elegans*, and the roles of LIM-4 in neuronal development. (A) The *unc-17* (VAChT) promoter region is systemically organized with distinct *cis*-regulatory DNA sequences for cell-type specific *tans*-acting factors, such as the SMB motif for LIM-4 in the SMB neurons, COE motif for UNC-3 in the A/B/AS-type or SAB motor neurons and the AIY motif for TTX-3/CHE-10 in the AIY interneurons. Additional terminal selector genes and their target *cis*-regulatory elements for remaining uncharacterized cholinergic cell-types need to be identified. (B) LIM-4 has distinct roles in neuronal development in a context-dependent manner.
(PDF)

## References

[1] Guillemot F. Spatial and temporal specification of neural fates by transcription factor codes. Development 2007;134: 3771–80. 17898002 10.1242/dev.006379

[2] Hobert O. Regulation of terminal differentiation programs in the nervous system. Annu Rev Cell Dev Biol 2011;27: 681–96. 10.1146/annurev-cellbio-092910-154226 21985672

[3] Deneris ES, Hobert O. Maintenance of postmitotic neuronal cell identity. Nat Neurosci 2014;17: 899–907. 10.1038/nn.3731 24929660 PMC4472461

[4] Li C, Kim K. Neuropeptides. WormBook 2008;1–36. 10.1895/wormbook.1.142.1 PMC2749236 18819171

[5] Duerr JS, Han HP, Fields SD, Rand JB. Identification of Major Classes of Cholinergic Neurons in the Nematode Caenorhabditis elegans. J Comp Neurol 2008;506: 398–408. 18041778 10.1002/cne.21551

[6] Altun-Gultekin Z, Andachi Y, Tsalik EL, Pilgrim D, Kohara Y, Hobert O. A regulatory cascade of three homeobox genes, ceh-10, ttx-3 and ceh-23, controls cell fate specification of a defined interneuron class in C. elegans. Development 2001;128: 1951–69. 11493519 10.1242/dev.128.11.1951

[7] Kratsios P, Stolfi A, Levine M, Hobert O. Coordinated regulation of cholinergic motor neuron traits through a conserved terminal selector gene. Nat Neurosci 2011;15: 205–14. 10.1038/nn.2989 22119902 PMC3267877

[8] Kratsios P, Pinan-Lucarré B, Kerk SY, Weinreb A, Bessereau JL, Hobert O. Transcriptional coordination of synaptogenesis and neurotransmitter signaling. Curr Biol 2015;25: 1282–95. 10.1016/j.cub.2015.03.028 25913400 PMC4465358

[9] Wenick AS, Hobert O. Genomic cis-regulatory architecture and trans-acting regulators of a single interneuron-specific gene battery in C. elegans. Dev Cell 2004;6: 757–70. 15177025 10.1016/j.devcel.2004.05.004

[10] Zhang F, Bhattacharya A, Nelson JC, Abe N, Gordon P, Lloret-Fernandez C, et al The LIM and POU homeobox genes ttx-3 and unc-86 act as terminal selectors in distinct cholinergic and serotonergic neuron types. Development 2014;141: 422–35. 10.1242/dev.099721 24353061 PMC3879818

[11] White JG, Southgate E, Thomson JN, Brenner S. The structure of the nervous system of the nematode Caenorhabditis elegans. Philos Trans R Soc Lond B Biol Sci 1986;314: 1–340. 22462104 10.1098/rstb.1986.0056

[12] Gray JM, Hill JJ, Bargmann CI. A circuit for navigation in Caenorhabditis elegans. Proc Natl Acad Sci U S A 2005;102: 3184–91. 15689400 10.1073/pnas.0409009101 PMC546636

[13] Kim K, Li C. Expression and regulation of an FMRFamide-related neuropeptide gene family in Caenorhabditis elegans. J Comp Neurol 2004;475: 540–50. 15236235 10.1002/cne.20189

[14] Sagasti A, Hobert O, Troemel ER, Ruvkun G, Bargmann CI. Alternative olfactory neuron fates are specified by the LIM homeobox gene lim-4. Genes Dev 1999;13: 1794–806. 10421632 10.1101/gad.13.14.1794 PMC316880

[15] Dawid IB1, Toyama R, Taira M. LIM domain proteins. C R Acad Sci III. 1995 3;318(3):295–306. 7788499

[16] Zheng X, Chung S, Tanabe T, Sze JY. Cell-type specific regulation of serotonergic identity by the C. elegans LIM-homeodomain factor LIM-4. Dev Biol 2005;286: 618–28. 16168406 10.1016/j.ydbio.2005.08.013

[17] Chou JH, Bargmann CI, Sengupta P. The Caenorhabditis elegans odr-2 gene encodes a novel Ly-6-related protein required for olfaction. Genetics 2001;157: 211–24. 11139503 10.1093/genetics/157.1.211 PMC1461492

[18] Colbert HA, Smith TL, Bargmann CI. OSM-9, a novel protein with structural similarity to channels, is required for olfaction, mechanosensation, and olfactory adaptation in Caenorhabditis elegans. J Neurosci 1997;17: 8259–69. 9334401 10.1523/JNEUROSCI.17-21-08259.1997 PMC6573730

[19] Alfonso A, Grundahl K, Duerr JS, Han HP, Rand JB. The Caenorhabditis elegans unc-17 gene: a putative vesicular acetylcholine transporter. Science 1993;261: 617–9. 8342028 10.1126/science.8342028

[20] Okuda T, Haga T, Kanai Y, Endou H, Ishihara T, Katsura I. Identification and characterization of the high-affinity choline transporter. Nat Neurosci 2000;3: 120–5. 10649566 10.1038/72059

[21] Pujol N, Torregrossa P, Ewbank JJ, Brunet JF. The homeodomain protein CePHOX2/CEH-17 controls antero-posterior axonal growth in C. elegans. Development 2000;127: 3361–71. 10887091 10.1242/dev.127.15.3361

[22] Kennerdell JR, Fetter RD, Bargmann CI. Wnt-Ror signaling to SIA and SIB neurons directs anterior axon guidance and nerve ring placement in C. elegans. Development 2009;136: 3801–10. 10.1242/dev.038109 19855022 PMC2861721

[23] Harfe BD, Fire A. Muscle and nerve-specific regulation of a novel NK-2 class homeodomain factor in Caenorhabditis elegans. Development 1998;125: 421–9. 9425137 10.1242/dev.125.3.421

[24] Sulston JE, Schierenberg E, White JG, Thomson JN. The embryonic cell lineage of the nematode Caenorhabditis elegans. Dev Biol 1983;100: 64–119. 6684600 10.1016/0012-1606(83)90201-4

[25] Brenner S. The genetics of Caenorhabditis elegans. Genetics 1974;77: 71–94. 4366476 10.1093/genetics/77.1.71 PMC1213120

[26] Lanjuin A, VanHoven MK, Bargmann CI, Thompson JK, Sengupta P. Otx/otd homeobox genes specify distinct sensory neuron identities in C. elegans. Dev Cell 2003;5: 621–33. 14536063 10.1016/s1534-5807(03)00293-4

[27] Fire A, Harrison SW, Dixon D. A modular set of lacZ fusion vectors for studying gene expression in Caenorhabditis elegans. Gene 1990;93: 189–198. 2121610 10.1016/0378-1119(90)90224-f

[28] Chang AJ, Chronis N, Karow DS, Marletta MA, Bargmann CI. A distributed chemosensory circuit for oxygen preference in C. elegans. PLoS Biol 2006;4: e274 16903785 10.1371/journal.pbio.0040274 PMC1540710

[29] Kim K, Kim R, Sengupta P. The HMX/NKX homeodomain protein MLS-2 specifies the identity of the AWC sensory neuron type via regulation of the ceh-36 Otx gene in C. elegans. Development 2010;137: 963–74. 10.1242/dev.044719 20150279 PMC2834459

[30] Serrano-Saiz E, Poole RJ, Felton T, Zhang F, De La Cruz ED, Hobert O. Modular control of glutamatergic neuronal identity in C. elegans by distinct homeodomain proteins. Cell 2013;155: 659–73. 10.1016/j.cell.2013.09.052 24243022 PMC3855022

[31] Bürglin TR. Homeodomain Sybtypes and Functional Diversity. A Handbook of Transcription Factors (ed. Hughes T.R..) Subcellular Biochemistry 2011;52 10.1007/978-90-481-9069-0_5

[32] Noyes MB, Christensen RG, Wakabayashi A, Stormo GD, Brodsky MH, Wolfe SA. Analysis of homeodomain specificities allows the family-wide prediction of preferred recognition sites. Cell 2008;133: 1277–89. 10.1016/j.cell.2008.05.023 18585360 PMC2478728

[33] Christensen RG, Enuameh MS, Noyes MB, Brodsky MH, Wolfe SA, Stormo GD. Recognition models to predict DNA-binding specificities of homeodomain proteins. Bioinformatics 2012;28: i84–9. 10.1093/bioinformatics/bts202 22689783 PMC3371834

[34] Flandin P, Zhao Y, Vogt D, Jeong J, Long J, Potter G, et al Lhx6 and Lhx8 coordinately induce neuronal expression of Shh that controls the generation of interneuron progenitors. Neuron 2011;70: 939–50. 10.1016/j.neuron.2011.04.020 21658586 PMC3153409

[35] Nokes EB, Van Der Linden AM, Winslow C, Mukhopadhyay S, Ma K, Sengupta P. Cis-regulatory mechanisms of gene expression in an olfactory neuron type in Caenorhabditis elegans. Dev Dyn 2009;238: 3080–92. 10.1002/dvdy.22147 19924784 PMC4078920

[36] Zhao Y, Marín O, Hermesz E, Powell A, Flames N, Palkovits M, et al The LIM-homeobox gene Lhx8 is required for the development of many cholinergic neurons in the mouse forebrain. Proc Natl Acad Sci U S A 2003;100: 9005–10. 12855770 10.1073/pnas.1537759100 PMC166428

[37] Mori T, Yuxing Z, Takaki H, Takeuchi M, Iseki K, Hagino S, et al The LIM homeobox gene, L3/Lhx8, is necessary for proper development of basal forebrain cholinergic neurons. Eur J Neurosci 2004;19: 3129–41. 15217369 10.1111/j.0953-816X.2004.03415.x

[38] Zhu P, Li H, Jin G, Tian M, Tan X, Shi J, et al LIM-homeobox gene Lhx8 promote the differentiation of hippocampal newborn neurons into cholinergic neurons in vitro. In Vitro Cell Dev Biol Anim 2013;49: 103–7. 10.1007/s11626-013-9582-8 23385486

[39] Shi J, Li H, Jin G, Zhu P, Tian M, Qin J, et al Lhx8 promote differentiation of hippocampal neural stem/progenitor cells into cholinergic neurons in vitro. In Vitro Cell Dev Biol Anim 2012;48: 603–9. 10.1007/s11626-012-9562-4 23150137

[40] Li H, Jin G, Zhu P, Zou L, Shi J, Yi X, et al Upregulation of Lhx8 increase VAChT expression and ACh release in neuronal cell line SHSY5Y. Neurosci Lett 2014;559: 184–8. 10.1016/j.neulet.2013.11.047 24316404

[41] Lopes FM, Schröder R, da Frota ML Jr, Zanotto-Filho A, Müller CB, Pires AS, et al Comparison between proliferative and neuron-like SH-SY5Y cells as an in vitro model for Parkinson disease studies. Brain Res 2010;1337: 85–94. 10.1016/j.brainres.2010.03.102 20380819

[42] Biedler JL, Roffler-Tarlov S, Schachner M, Freedman LS. Multiple neurotransmitter synthesis by human neuroblastoma cell lines and clones. Cancer Res 1978;38: 3751–7. 29704

[43] Kovalevich J, Langford D. Considerations for the use of SH-SY5Y neuroblastoma cells in neurobiology. Methods Mol Biol 2013;1078: 9–21. 10.1007/978-1-62703-640-5_2 23975817 PMC5127451

[44] Danks K, Wade JA, Batten TF, Walker JH, Ball SG, Vaughan PF. Redistribution of F-actin and large dense-cored vesicles in the human neuroblastoma SH-SY5Y in response to secretagogues and protein kinase Calpha activation. Brain Res Mol Brain Res. 1999 2 5;64(2):236–45. 9931495 10.1016/s0169-328x(98)00325-8

[45] Hobert O. Regulatory logic of neuronal diversity: terminal selector genes and selector motifs. Proc Natl Acad Sci U S A 2008;105: 20067–71. 10.1073/pnas.0806070105 19104055 PMC2629285

[46] Wightman B, Ebert B, Carmean N, Weber K, Clever S. The C. elegans nuclear receptor gene fax-1 and homeobox gene unc-42 coordinate interneuron identity by regulating the expression of glutamate receptor subunits and other neuron-specific genes. Dev Biol 2005;287: 74–85. 16183052 10.1016/j.ydbio.2005.08.032

[47] Palmer R, Inoue T, Sherwood DR, Jiang LI, Sternberg PW. Caenorhabditis elegans cog-1 locus encodes GTX/Nkx6.1 homeodomain proteins and regulates multiple aspects of reproductive system development. Dev Biol 2002;252: 202–13. 12482710 10.1006/dbio.2002.0850

[48] Sarafi-Reinach TR, Melkman T, Hobert O, Sengupta P. The lin-11 LIM homeobox gene specifies olfactory and chemosensory neuron fates in C. elegans. Development 2001;128: 3269–81. 11546744 10.1242/dev.128.17.3269

[49] Sarin S, Antonio C, Tursun B, Hobert O. The C. elegans Tailless/TLX transcription factor nhr-67 controls neuronal identity and left/right asymmetric fate diversification. Development 2009;136: 2933–44. 10.1242/dev.040204 19641012 PMC2723065

[50] Bargmann CI, Horvitz HR. Chemosensory neurons with overlapping functions direct chemotaxis to multiple chemicals in C. elegans. Neuron 1991;7: 729–42. 1660283 10.1016/0896-6273(91)90276-6

[51] Holland PW, Takahashi T. The evolution of homeobox genes: Implications for the study of brain development. Brain Res Bull 2005;66: 484–90. 16144637 10.1016/j.brainresbull.2005.06.003

[52] Hobert O, Westphal H. Functions of LIM-homeobox genes. Trends Genet 2000;16: 75–83. 10652534 10.1016/s0168-9525(99)01883-1

[53] Srivastava M, Larroux C, Lu DR, Mohanty K, Chapman J, Degnan BM, et al Early evolution of the LIM homeobox gene family. BMC Biol 2010;8: 4 10.1186/1741-7007-8-4 20082688 PMC2828406

[54] Cho HH, Cargnin F, Kim Y, Lee B, Kwon RJ, Nam H, et al Isl1 directly controls a cholinergic neuronal identity in the developing forebrain and spinal cord by forming cell type-specific complexes. PLoS Genet 2014;10: e1004280 10.1371/journal.pgen.1004280 24763339 PMC3998908

[55] Lopes R, Verhey van Wijk N, Neves G, Pachnis V. Transcription factor LIM homeobox 7 (Lhx7) maintains subtype identity of cholinergic interneurons in the mammalian striatum. Proc Natl Acad Sci U S A 2012;109: 3119–24. 10.1073/pnas.1109251109 22315402 PMC3286949

[56] Fragkouli A, van Wijk NV, Lopes R, Kessaris N, Pachnis V. LIM homeodomain transcription factor-dependent specification of bipotential MGE progenitors into cholinergic and GABAergic striatal interneurons. Development 2009;136: 3841–51. 10.1242/dev.038083 19855026 PMC2766344

[57] Vogt D, Hunt RF, Mandal S, Sandberg M, Silberberg SN, Nagasawa T, et al Lhx6 directly regulates Arx and CXCR7 to determine cortical interneuron fate and laminar position. Neuron 2014;82: 350–64. 10.1016/j.neuron.2014.02.030 24742460 PMC4261952

[58] Chao MY, Komatsu H, Fukuto HS, Dionne HM, Hart AC. Feeding status and serotonin rapidly and reversibly modulate a Caenorhabditis elegans chemosensory circuit. Proc Natl Acad Sci U S A 2004;101: 15512–7. 15492222 10.1073/pnas.0403369101 PMC524441

[59] L’Etoile ND, Bargmann CI. Olfaction and odor discrimination are mediated by the C. elegans guanylyl cyclase ODR-1. Neuron 2000;25: 575–86. 10774726 10.1016/s0896-6273(00)81061-2

[60] Hedgecock EM, White JG. Polyploid tissues in the nematode Caenorhabditis elegans. Dev Biol 1985;107: 128–33. 2578115 10.1016/0012-1606(85)90381-1

