# Supplementary material for: The Evolutionarily Conserved LIM Homeodomain Protein LIM-4/LHX6 Specifies the Terminal Identity of a Cholinergic and Peptidergic C. elegans Sensory/Inter/Motor Neuron-Type
Source: PLoS Genet. 2015 Aug 25;11(8):e1005480. doi: 10.1371/journal.pgen.1005480 (PMC4549117; doi:10.1371/journal.pgen.1005480)
Supplement: S1 Table — Expression pattern of flp-12p::gfp reporter constructs in the SMB neurons was observed in wild-type (WT) or lim-4 (lsk3, lsk5, yn19) mutant animals at L1 larval stage (L1). Expression was observed at 630x. Strong, weak or no expression is defined as GFP expression observed in both cell bodies and processes, observed in only cell bodies, or not observed either in cell bodies or processes, respectively. n≥50. (PDF) [file pgen.1005480.s001.pdf]

S1 Table. Expression pattern of the *flp-12* in *lim-4* mutant animals at L1 stage

Reporter construct	Genotype	% animals showing GFP expression in SMB (L1)		
		No	Weak	Strong
*flp-12*p::*gfp* *(ynIs82)*	WT	0	0	100
	lsk3	20	70	10
*flp-12*p::*gfp* *(ynIs25)*	WT	0	0	100
	yn19	100	0	0
	lsk5	100	0	0
