# Supplementary material for: The Evolutionarily Conserved LIM Homeodomain Protein LIM-4/LHX6 Specifies the Terminal Identity of a Cholinergic and Peptidergic C. elegans Sensory/Inter/Motor Neuron-Type
Source: PLoS Genet. 2015 Aug 25;11(8):e1005480. doi: 10.1371/journal.pgen.1005480 (PMC4549117; doi:10.1371/journal.pgen.1005480)
Supplement: S2 Table — Lipophilic dye DiD was used to observe dye-filling in the AWB neurons of lim-4 mutant animals (ky403, yn19, lsk3, lsk5). In lim-4 (ky403, lsk5) null mutants, the AWB neurons are not dye-filled, while the ADF neurons are ectopically dye-filled [16]. Dye staining in adult animals was observed at 400x. n≥30. (PDF) [file pgen.1005480.s002.pdf]

S2 Table. Dye-filling defects in the AWB neurons of *lim-4* mutant animals

Genotype	% animals showing dye-filling in AWB/ADF
WT	100
ky403	23
yn19	3
lsk3	0
lsk5	57
