# Supplementary material for: The Evolutionarily Conserved LIM Homeodomain Protein LIM-4/LHX6 Specifies the Terminal Identity of a Cholinergic and Peptidergic C. elegans Sensory/Inter/Motor Neuron-Type
Source: PLoS Genet. 2015 Aug 25;11(8):e1005480. doi: 10.1371/journal.pgen.1005480 (PMC4549117; doi:10.1371/journal.pgen.1005480)
Supplement: S3 Table — Lipophilic dye DiD was used to observe dye-filling in the AWB/ADF neurons of lim-4 mutant animals. Dye staining in adult animals was observed at 400x. n≥30. (PDF) [file pgen.1005480.s003.pdf]

S3 Table. Dye-filling defects of *lim-4* mutants are partially rescued by temporal expression of LIM-4

Genotype	Extrachromosomal array		% animals showing dye-filling in AWB/ADF
WT	-	No Heat shock	100
lim-4(ky403)	-	No Heat shock	23
	-	Heat shock	17
	*Ex[lim-4*p*Δ3::lim-4cDNA]*	No Heat shock	33
	Ex[hsp::lim-4cDNA]	Heat shock	77
	Ex[hsp::LHX6cDNA]	Heat shock	53
	Ex[hsp::LHX8cDNA]	Heat shock	50
