# Supplementary material for: The Evolutionarily Conserved LIM Homeodomain Protein LIM-4/LHX6 Specifies the Terminal Identity of a Cholinergic and Peptidergic C. elegans Sensory/Inter/Motor Neuron-Type
Source: PLoS Genet. 2015 Aug 25;11(8):e1005480. doi: 10.1371/journal.pgen.1005480 (PMC4549117; doi:10.1371/journal.pgen.1005480)
Supplement: S4 Table — Expression pattern of flp-12p::gfp or lim-4p::gfp reporter constructs in the SMB neurons was observed in wild-type (WT), fax-1 (gm83) or cog-1(sy275) mutant animals. Expression was observed at 400x. Strong, weak or no expression is defined as GFP expression observed in both cell bodies and processes, observed in only cell bodies, or not observed either in cell bodies or processes, respectively. n≥50. (PDF) [file pgen.1005480.s004.pdf]

S4 Table. The expression of the *flp-12* or *lim-4* in the SMB neurons is not altered in *fax-1* or *cog-1* mutant animals

Reporter construct	Genotype	% animals showing GFP expression in SMB		
		No	Weak	Strong
*Ex[flp-12*pΔ*1::gfp]*	WT	0	0	100
	fax-1(gm83)	0	0	100
	cog-1(sy275)	0	0	100
*lim-4*p*::gfp(oyIs35)*	WT	0	0	100
	fax-1(gm83)	0	0	100
	cog-1(sy275)	0	0	100
