# Supplementary material for: The Evolutionarily Conserved LIM Homeodomain Protein LIM-4/LHX6 Specifies the Terminal Identity of a Cholinergic and Peptidergic C. elegans Sensory/Inter/Motor Neuron-Type
Source: PLoS Genet. 2015 Aug 25;11(8):e1005480. doi: 10.1371/journal.pgen.1005480 (PMC4549117; doi:10.1371/journal.pgen.1005480)
Supplement: S5 Table — (PDF) [file pgen.1005480.s005.pdf]

S5 Table. Transgenes and strains used in this study

**Transgene**	***Genotype***	**Strain**
*ace-2*p*::gfp*	*otEx4432*	OH10850
*acr-2*p*::gfp*	*juIs14*	OH10851
*acr-5*p*::gfp*	*wdEx75*	NC216
*acr-14*p*::gfp*	*wdEx455*	NC1013
*ceh-17*p*::dsRed*	*lskEx239*	KHK246
	lskEx240	KHK247
*ceh-24*p*::gfp*	*ccIs4595*	PD4595
*ceh-36*p*::lim-4cDNA*	*lskEx369*	KHK383
	lskEx406	KHK409
*cho-1*p*::gfp*	*otIs323*	OH10101
cho-1^{fosmid}*::yfp*	*otIs534*	
cho-1^{fosmid}*::yfp; eat-4*^{fosmid}*::mChOpti*	*otIs534; otIs518*	
cog-1	*sy275*	PS2571
*cog-1*p*::gfp*	*syIs63*	PS3662
*eat-4*pΔ*5::lim-4cDNA*	*lskEx374*	KHK388
	lskEx375	KHK389
	lskEx376	KHK390
fax-1	*gm83*	NG83
*flp-7*p^{-2446}*::gfp*	*ynIs66*	NY2066
*flp-7*p^{-2446}*-SMBmotif*$^{-353}$*::gfp*	*lskEx156*	KHK160
	lskEx157	KHK161
*flp-12*p^{-2652}*::gfp*	*ynIs25*	NY2025
	ynIs82	NY2082
*flp-12*p^{-1547}*::gfp*	*lskEx3*	KHK3
	lskEx4	KHK4
*flp-12*p^{-523}*::gfp*	*lskEx1*	KHK1
	lskEx2	KHK2
*flp-12*p^{-523}*-MUT*$^{-501}$*::gfp*	*lskEx97*	KHK101
	lskEx98	KHK102
*flp-12*p^{-523}*-MUT*$^{-441}$*::gfp*	*lskEx99*	KHK103
	lskEx100	KHK104
*flp-12*p^{-523}*-MUT*$^{-429}$*::gfp*	*lskEx101*	KHK105
	lskEx102	KHK106
*flp-12*p^{-523}*-MUT*$^{-429}$*::gfp*	*lskEx103*	KHK107
	lskEx104	KHK108
*flp-12*p^{-523}*-MUT*$^{-501-441-429-392}$*::gfp*	*lskEx87*	KHK91
	lskEx88	KHK92
*flp-12*p^{-483}*::gfp*	*lskEx91*	KHK95
	lskEx92	KHK96
*flp-12*p^{-436}*::gfp*	*lskEx93*	KHK97
	lskEx94	KHK98

*flp-12*p^{-393}*::gfp*	*lskEx95*	KHK99
	lskEx96	KHK100
*flp-12*p^{-393}*-MUT*$^{-370-361}$*::gfp*	*lskEx105*	KHK109
	lskEx106	KHK110
*flp-12*p^{-393}*-MUT*$^{-377}$*::gfp*	*lskEx107*	KHK111
	lskEx108	KHK112
*flp-12*p^{-393}*-MUT*$^{-383}$*::gfp*	*lskEx109*	KHK113
	lskEx110	KHK114
*flp-12*p^{-393}*-MUT*$^{-363}$*::gfp*	*lskEx111*	KHK115
	lskEx112	KHK116
*flp-12*p^{-393}*-MUT*$^{-341}$*::gfp*	*lskEx158*	KHK163
	lskEx159	KHK164
*flp-12*p^{-339}*::gfp*	*lskEx13*	KHK13
	lskEx14	KHK14
*flp-12*p^{-312}*::gfp*	*lskEx15*	KHK15
	lskEx16	KHK16
*flp-12*p^{-312}*-MUT*$^{-190}$*::gfp*	*lskEx77*	KHK81
	lskEx78	KHK82
*flp-12*p^{-312}*-MUT*$^{-193}$*::gfp*	*lskEx79*	KHK83
*flp-12*p^{-312}*-MUT*$^{-167}$*::gfp*	*lskEx81*	KHK85
	lskEx82	KHK86
*flp-12*p^{-312}*-MUT*$^{-243}$*::gfp*	*lskEx83*	KHK87
	lskEx84	KHK88
*flp-12*p^{-312}*-MUT*$^{-232}$*::gfp*	*lskEx85*	KHK89
	lskEx86	KHK90
*flp-12*p^{-312}*-MUT*$^{-243-232}$*::gfp*	*lskEx89*	KHK93
	lskEx90	KHK94
*flp-12*p^{-162}*::gfp*	*lskEx20*	KHK20
	lskEx21	KHK21
*flp-12*p^{-34}*::gfp*	*lskEx22*	KHK22
	lskEx23	KHK23
*flp-12*p*::mCherry*	*lskEx294*	KHK301
*flp-22*p*::gfp*	*lskEx248*	KHK255
	lskEx249	KHK256
hsp16.2::human LHX6 cDNA	*lskEx184*	KHK189
	lskEx186	KHK191
	lskEx222	KHK229
	lskEx223	KHK230
hsp16.2::human LHX8 cDNA	*lskEx227*	KHK234
	lskEx229	KHK236
	lskEx267	KHK274
	lskEx268	KHK275
hsp16.2::lim-4 cDNA	*lskEx51*	KHK51

	lskEx52	KHK52
lim-4	*ky403*	CX3937
	lsk1	KHK452
	lsk2	KHK453
	lsk3	KHK454
	lsk4	KHK455
	lsk5	KHK456
	lsk6	KHK459
	lsk7	KHK461
	yn19	NY225
*lim-4*p^{-3583}*::gfp*	*oyIs35*	PY1958
	lskEx265	KHK272
	lskEx266	KHK273
*lim-4*p$^{-3379-1923}$*::gfp*	*lskEx32*	KHK32
	lskEx33	KHK33
*lim-4*p$^{-3379-1525}$*::gfp*	*lskEx60*	KHK60
	lskEx61	KHK61
*lim-4*p$^{-3379-1525}$*-MUT*$^{-64}$*::gfp*	*lskEx272*	KHK279
*lim-4*p$^{-3379-999}$*::gfp* (*lim-4*pΔ*3::gfp*)	*lskEx55*	KHK55
	lskEx56	KHK56
*lim-4*p$^{-3379-999}$*::lim-4cDNA* (*lim-4*pΔ*3::lim-4cDNA*)	*lskEx224*	KHK231
	lskEX225	KHK232
	lskEx226	KHK233
*lim-4*p$^{-3379-999}$*::mCherry* (*lim-4*pΔ*3::mCherry*)	*lskEx175*	KHK180
*lim-4*p$^{-3477-461}$*::gfp*	*lskEx11*	KHK11
	lskEx12	KHK12
*lim-4*p$^{-2288-735}$*::gfp*	*lskEx7*	KHK7
	lskEx8	KHK8
*lim-4*p$^{-1793-275}$*::gfp*	*lskEx38*	KHK38
	lskEx39	KHK39
*lim-4*p$^{-1793-247}$*::gfp*	*lskEx41*	KHK41
	lskEx42	KHK42
*lim-4*p$^{-1793-38}$*::gfp*	*lskEx46*	KHK46
	lskEx47	KHK47
*lim-4*p$^{-999-38}$*::gfp*	*lskEx129*	KHK133
	lskEx74	KHK78
*lim-4*p$^{-999-247}$*::gfp*	*lskEx134*	KHK139
	lskEx135	KHK140
*lim-4*p^{-3327}*::gfp*	*lskEx138*	KHK143
	lskEx141	KHK146
*lim-4*p^{-1923}*::gfp*	*lskEx144*	KHK149
	lskEx145	KHK150

*lim-4*p$^{-3379-1922}$*::gfp*	*lskEx148*	KHK153
	lskEx149	KHK154
*lim-4*p^{-82}*::gfp*	*lskEx123*	KHK127
	lskEx124	KHK128
	lskEx125	KHK129
	lskEx126	KHK130
*lim-4*p^{-82}-*MUT*$^{-53}$*::gfp*	*lskEx271*	KHK278
*lim-4*p^{-82}-*MUT*$^{-64}$*::gfp*	*lskEx146*	KHK151
	lskEx147	KHK152
*lim-4*p^{-38}*::gfp*	*lskEx270*	KHK277
*lim-4*p^{-999}*::gfp*	*lskEx194*	KHK201
*odr-1*p*::dsRed*	*lskEx370*	KHK384
	lskEx372	KHK386
	lskEx373	KHK387
*odr-1*p*::lim-4cDNA*	*lskEx139*	KHK143
	lskEx140	KHK145
*odr-2*p^{-2443}*::gfp*	*lskEx115*	KHK119
	lskEx116	KHK121
*odr-2*p^{-607}*::gfp*	*lskEx117*	KHK121
	lskEx118	KHK122
*odr-2*p^{-607}-*MUT*$^{-570}$*::gfp*	*lskEx165*	KHK170
	lskEx166	KHK171
*odr-2*p^{-607}-*MUT*$^{-548}$*::gfp*	*lskEx167*	KHK172
	lskEx168	KHK173
*odr-2*p^{-527}*::gfp*	*lskEx161*	KHK166
	lskEx162	KHK167
*odr-2*p^{-377}*::gfp*	*lskEx163*	KHK168
	lskEx164	KHK169
*odr-2*p^{-300}*::gfp*	*lskEx119*	KHK123
	lskEx120	KHK124
*rgef-1*p*::gfp*	*evIs111*	NW1229
*trp-1*p*::gfp*	*kyIs123*	OH1358
*unc-17*p^{-4410}*::gfp*	*vsIs48*	LX929
	lskEx292	KHK299
	lskEx295	KHK302
*unc-17*p^{-4410}-*MUT*$^{-4270}$*::gfp*	*lskEx275*	KHK282
	lskEx276	KHK283
*unc-17*p^{-4175}*::gfp*	*lskEx250*	KHK257
*unc-17*p^{-2820}*::gfp*	*lskEx217*	KHK224
*unc-17*p^{-2242}*::gfp*	*lskEx219*	KHK226
*unc-42*p*::gfp*	*gmEx104*	NG2591
*unc-119*p*::gfp*	*otIs45*	OH441
