# Supplementary figures and images for: The Evolutionarily Conserved LIM Homeodomain Protein LIM-4/LHX6 Specifies the Terminal Identity of a Cholinergic and Peptidergic C. elegans Sensory/Inter/Motor Neuron-Type
Source: PLoS Genet. 2015 Aug 25;11(8):e1005480. doi: 10.1371/journal.pgen.1005480 (PMC4549117; doi:10.1371/journal.pgen.1005480)

*flp-12p::gfp(ynIs82)*

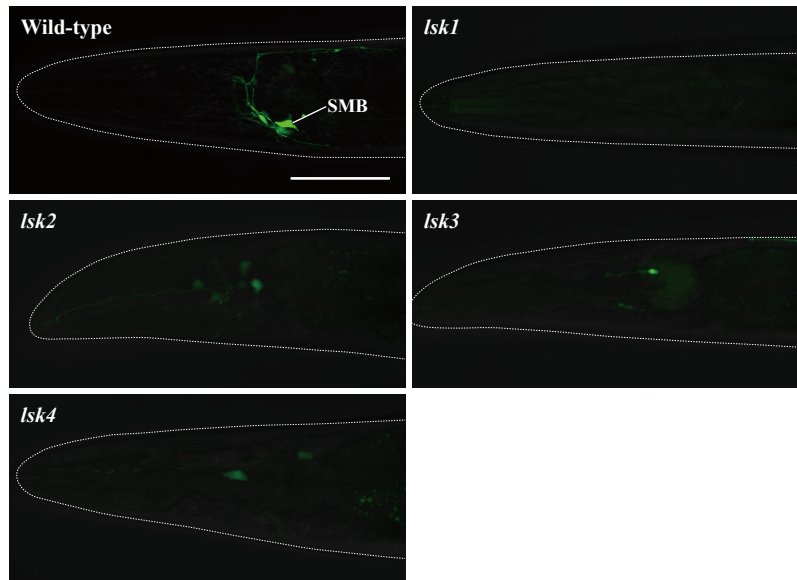

*flp-12p::gfp(ynIs25)*

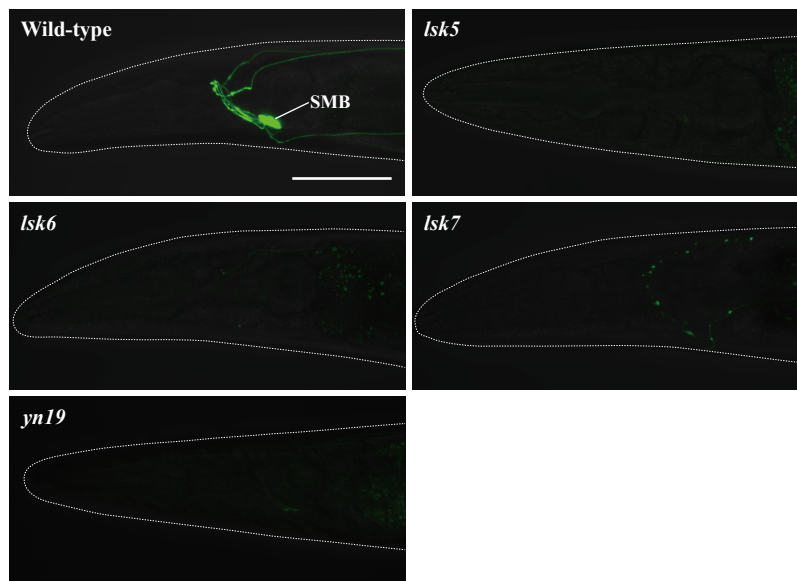

Supplement: S1 Fig — Two integrated strains expressing a flp-12p::gfp reporter (ynIs82 or ynIs25) were used for EMS mutagenesis screens. Note that ynIs82 integrated strains exhibit variable and weak GFP expression in a few neurons of the head. Anterior is at left in all images. Scale bars: 50 μm. (PDF) [file pgen.1005480.s006.pdf]

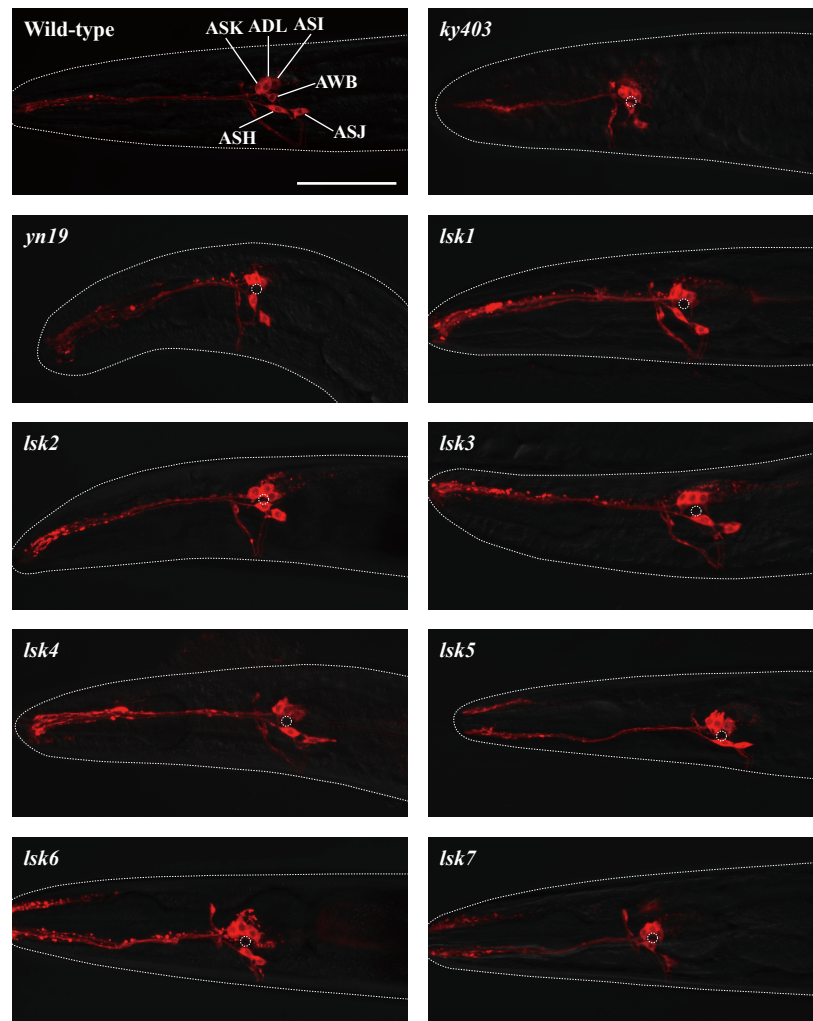

Supplement: S2 Fig — The AWB neurons and additional five pairs of amphid neurons (ASK, ADL, ASI, ASH, ASJ) in the head of wild-type animals fill with lipophilic dye DiD [60]. In ky403 and eight newly identified lim-4 alleles, the AWB neurons fail to be dye-filled. Images are derived from z-stacks of confocal microscopy images taken for left-side amphid neurons. Dashed circles indicate position of the AWB neurons. Anterior is at left in all images. Quantitative analysis of these phenotypes is shown in S2 Table. Scale bar: 50 μm. (PDF) [file pgen.1005480.s007.pdf]

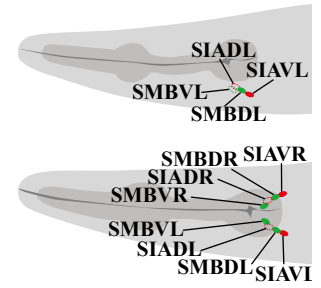

Supplement: S3 Fig — Shown are the images of expression of flp-12p::gfp (SMB) or ceh-17p::dsRed (SIA), the merged images, or schematic drawing of cell bodies in the merged images. Top images are from lateral view and bottom images are from ventral view of head of worms. Although positions of the cell bodies of SMBs or SIAs are variable, we consistently observed that the cell bodies of SMBDL/R and SIAVL/R are located immediately adjacent to each other at the same focal plane in all tested animals (n>30). Therefore, we identified SMBDL/R cell bodies via Nomarski optics by comparing with expression of ceh-17p::dsRed in the cell body of SIAVL/R. Images are derived from z-stacks of confocal microscopy images while images in the upper-left boxed regions are single focal plane confocal microscopy images. Anterior is at left in all images. Scale bar: 50 μm. (PDF) [file pgen.1005480.s008.pdf]

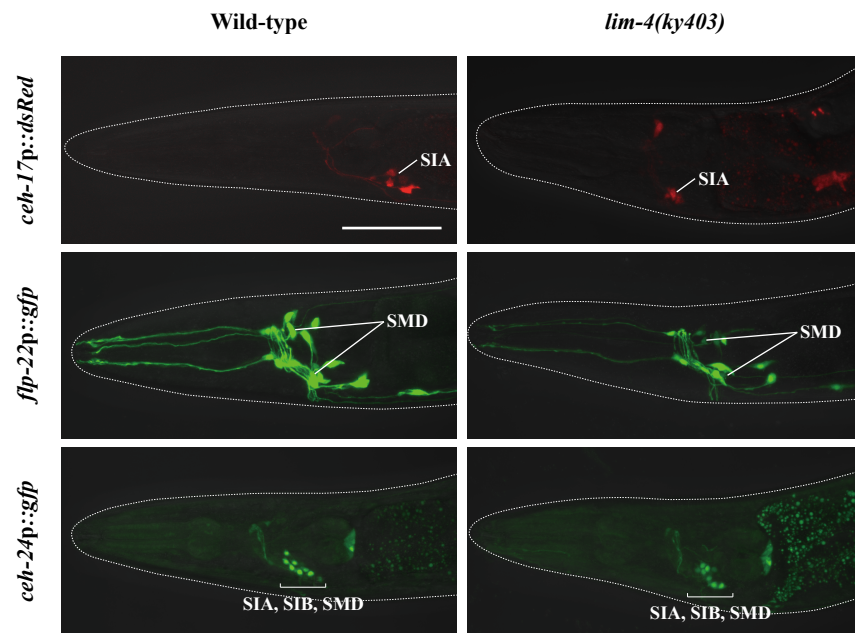

Supplement: S4 Fig — Expression of the indicated reporter constructs is shown in wild-type (left column) or lim-4(ky403) mutant (right column) animals. Expression of the ceh-24p::gfp reporter is detected in about 8 cells bodies in the head of worms including the SIA, SIB, and SMD neurons but not the SMB neurons. Images are derived from z-stacks of confocal microscopy images. Anterior is to the left. Scale bar: 50 μm. (PDF) [file pgen.1005480.s009.pdf]

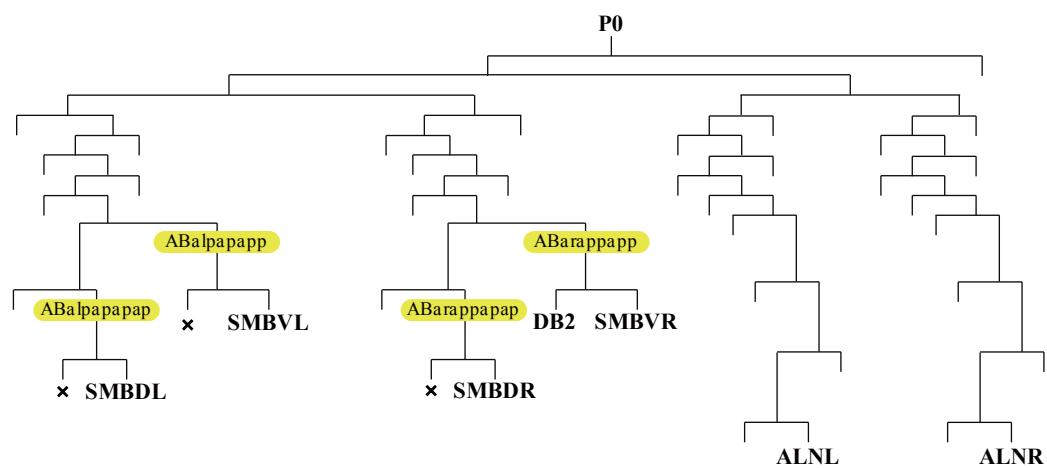

Supplement: S5 Fig — Precursor cells of the SMB neurons are shown in yellow circles. x indicates the programmed cell death. Heat shocks to transgenic animals expressing hsp::lim-4 cDNA transgene induced flp-12 expression in the ALN neurons (see Fig 5A). (PDF) [file pgen.1005480.s010.pdf]

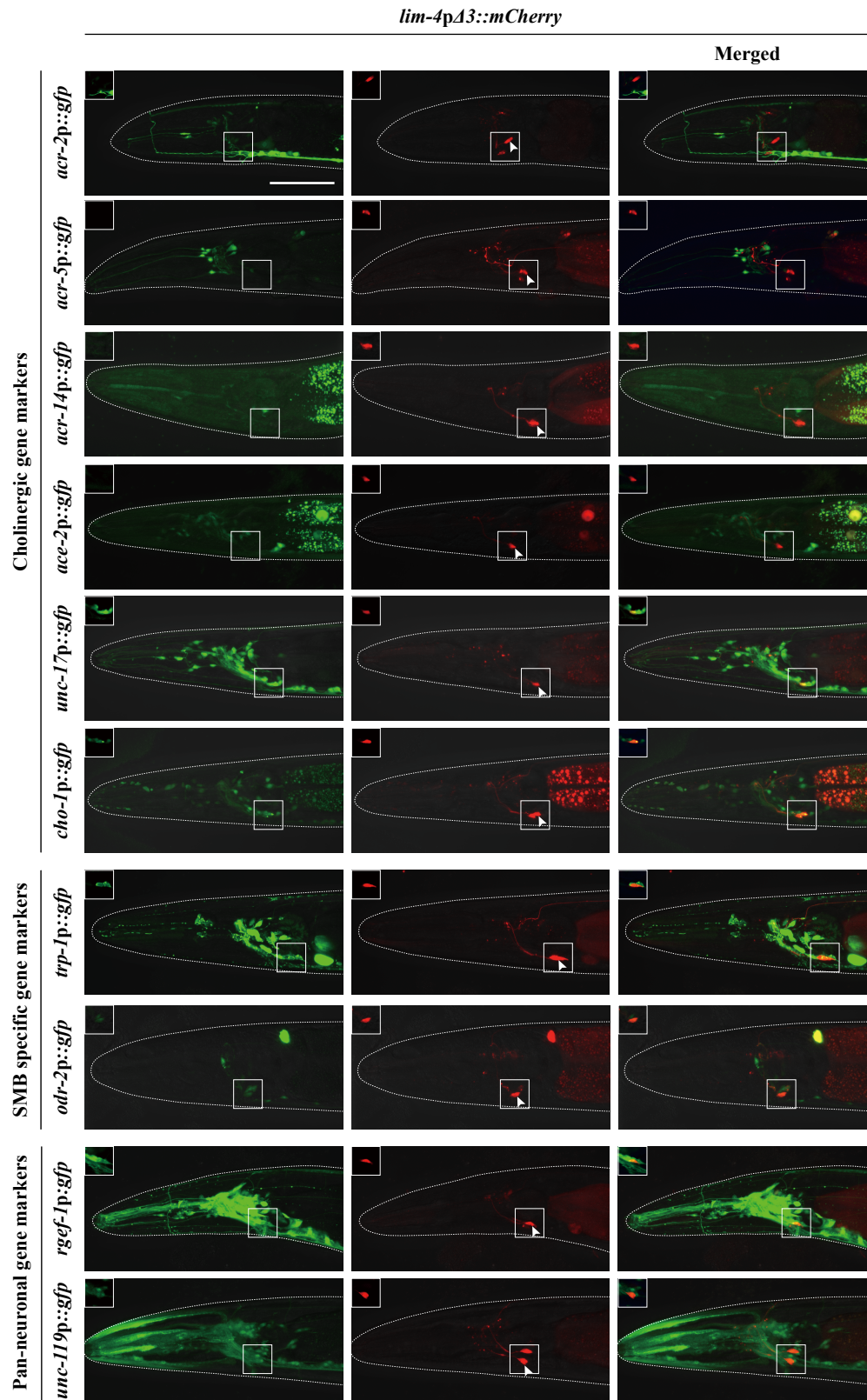

Supplement: S6 Fig — Expression of gfp reporter constructs under the control of cholinergic (unc-17, cho-1), SMB-specific (trp-1, odr-2) or pan-neuronal (rgef-1, unc-119) gene promoter is overlapped with expression of the lim-4pΔ3::mCherry reporter in the SMB neurons of wild-type animals. Expression of acr-2, acr-5, acr-14(AChRs), or ace-2 (AChE) cholinergic marker is not detected in the SMB neurons. Expression of the indicated reporter constructs and of the lim-4pΔ3::mCherry reporter is shown in left or middle column, respectively, and the merged images are shown in right column. Images are derived from z-stacks of confocal microscopy images while images in the upper-left boxed regions are single focal plane confocal microscopy images. Anterior is at left in all images. Scale bar: 50 μm. (PDF) [file pgen.1005480.s011.pdf]

*flp-12p::gfp(ynIs25);*  
*Ex[ceh-36p::lim-4cDNA;odr-1p::dsRed]*

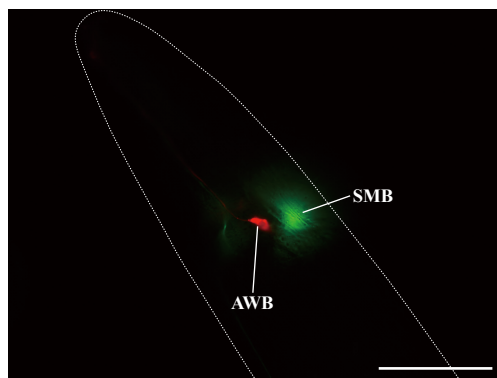

Supplement: S7 Fig — ceh-36 gene promoter drives LIM-4 expression in the AWC and ASE neurons [26]. Ex[odr-1p::dsRed] transgenic animals express dsRed in AWC and AWB [59]. Anterior is at left in all images. Scale bar: 50 μm. (PDF) [file pgen.1005480.s012.pdf]

*cho-1<sup>fosmid</sup>::yfp;eat-4<sup>fosmid</sup>::mChOpti*

Wild-type

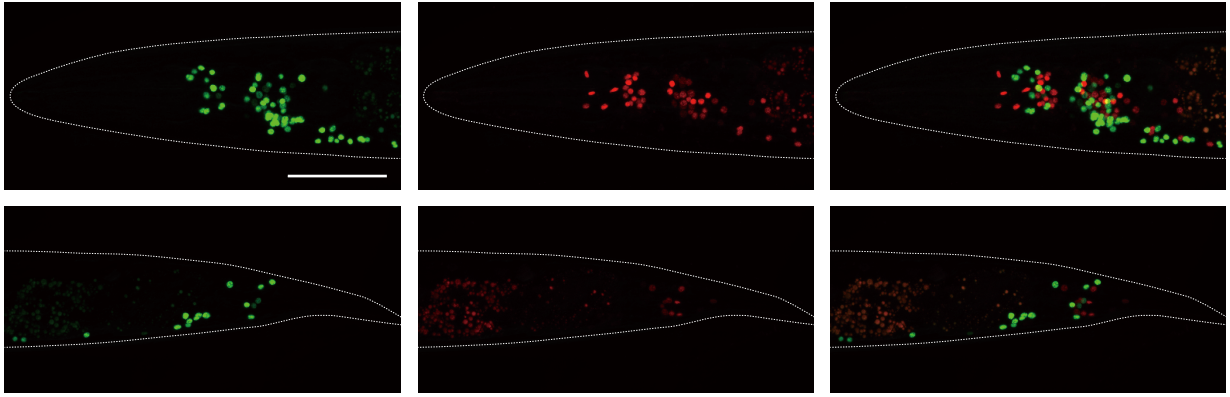

Ex[*eat-4p*Δ5::lim-4cDNA]

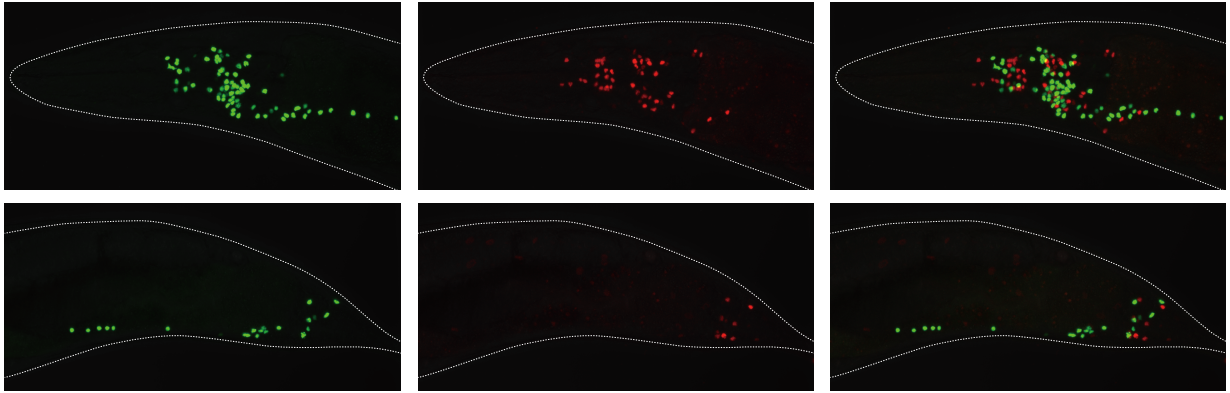

Supplement: S8 Fig — eat-4pΔ5 promoter drives LIM-4 expression in 11 out of 38 glutamatergic neuron types [30]. Images are derived from z-stacks of confocal microscopy images. Anterior is at left in all images. Scale bar: 50 μm. (PDF) [file pgen.1005480.s013.pdf]

**ARROWHEAD**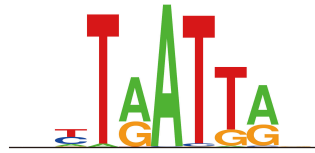**LHX6**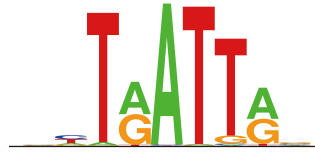**LHX8**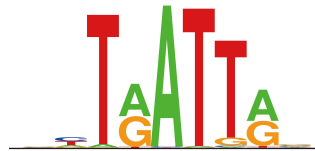

Supplement: S9 Fig — The binding sites are derived from a web based tool, PreMoTF (http://stormo.wustl.edu/PreMoTF) [33]. (PDF) [file pgen.1005480.s014.pdf]

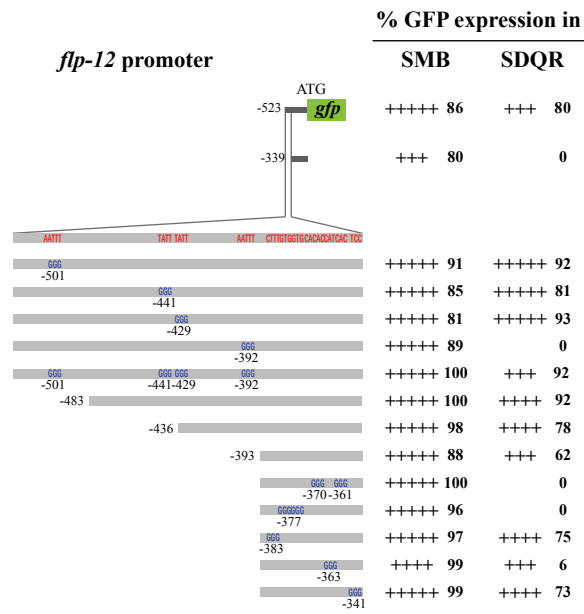

Supplement: S10 Fig — A cis-regulatory sequence for the SMB neurons was not identified in this promoter region. The percentage of transgenic animals expressing each gfp reporter construct in the indicated neurons is shown. Strength of GFP expression is indicated by the number of + symbols. Point mutated nucleotides are indicated as wild-type in red line. At least two independent extrachromosomal lines for each construct were examined. n≥50 for each. (PDF) [file pgen.1005480.s015.pdf]

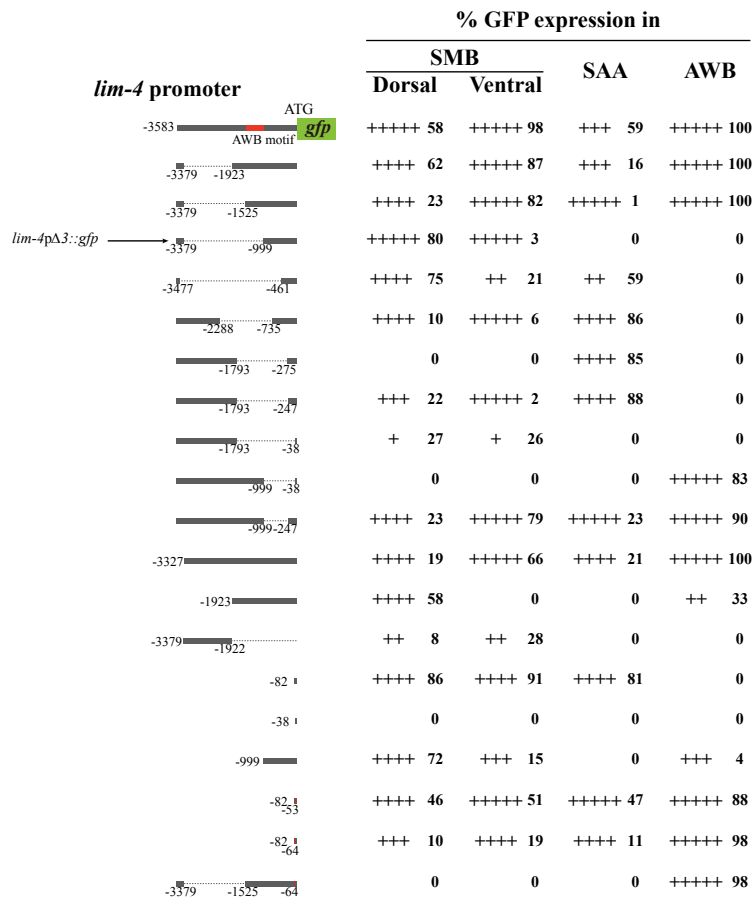

Supplement: S11 Fig — The percentage of transgenic animals expressing each gfp reporter construct in the indicated neurons is shown. Strength of GFP expression is indicated by the number of + symbols. Deleted regions in the promoter are indicated as a dotted line. Mutated nucleotides are indicated as wild-type in red line. The cis-regulatory sequence for the AWB expression of lim-4 is indicated. At least two independent extrachromosomal lines for each construct were examined. n ≥50 for each. (PDF) [file pgen.1005480.s016.pdf]

**A**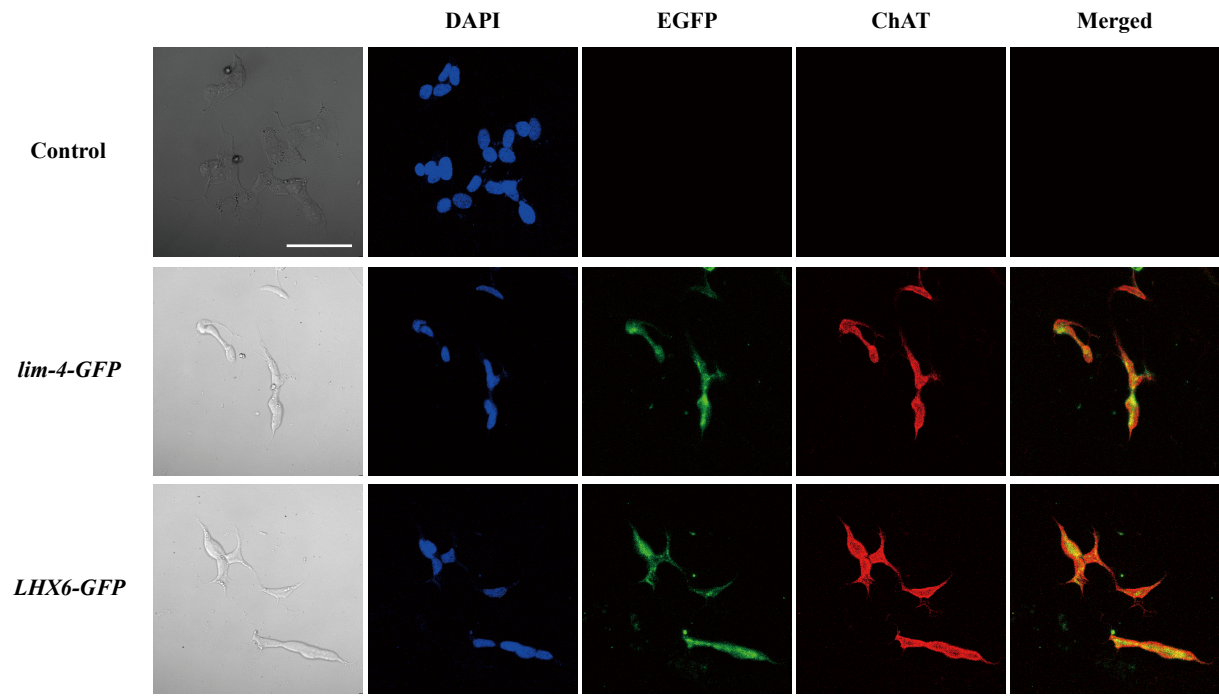**B**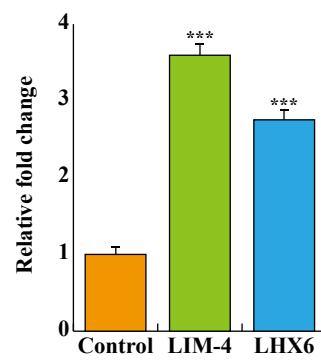

Supplement: S13 Fig — (A) Confocal images of SH-SY5Y human neuroblastoma cell line transfected by C. elegans lim-4 or human LHX6 and immunostained with ChAT antibodies. Scale bar: 50 μm. (B) Levels of ChAT transcripts are increased in SH-SY5Y human neuroblastoma cell line transfected by C. elegans lim-4 or human LHX6. The relative fold change to housekeeping gene GAPDH is shown. *** indicates significantly different from control (untransfected cells) (p<0.001). (PDF) [file pgen.1005480.s018.pdf]

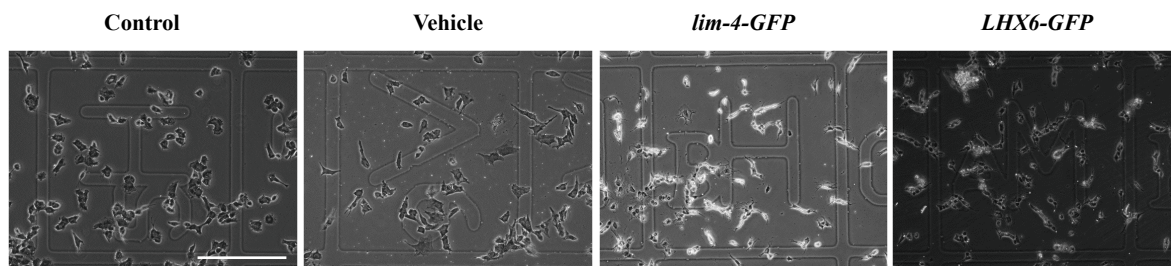

Supplement: S14 Fig — Scale bar: 500 μm (PDF) [file pgen.1005480.s019.pdf]

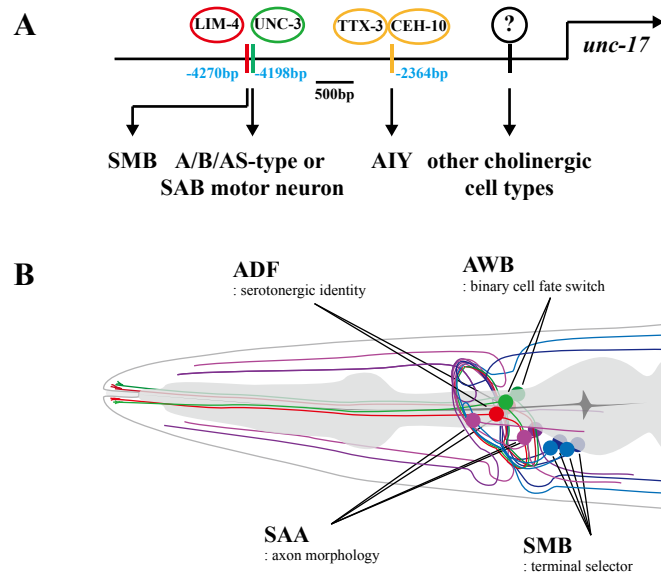

Supplement: S15 Fig — (A) The unc-17 (VAChT) promoter region is systemically organized with distinct cis-regulatory DNA sequences for cell-type specific tans-acting factors, such as the SMB motif for LIM-4 in the SMB neurons, COE motif for UNC-3 in the A/B/AS-type or SAB motor neurons and the AIY motif for TTX-3/CHE-10 in the AIY interneurons. Additional terminal selector genes and their target cis-regulatory elements for remaining uncharacterized cholinergic cell-types need to be identified. (B) LIM-4 has distinct roles in neuronal development in a context-dependent manner. (PDF) [file pgen.1005480.s020.pdf]
