# Supplementary material for: The Evolutionarily Conserved LIM Homeodomain Protein LIM-4/LHX6 Specifies the Terminal Identity of a Cholinergic and Peptidergic C. elegans Sensory/Inter/Motor Neuron-Type
Source: PLoS Genet. 2015 Aug 25;11(8):e1005480. doi: 10.1371/journal.pgen.1005480 (PMC4549117; doi:10.1371/journal.pgen.1005480)

Kim & Yeon_S12_Fig.

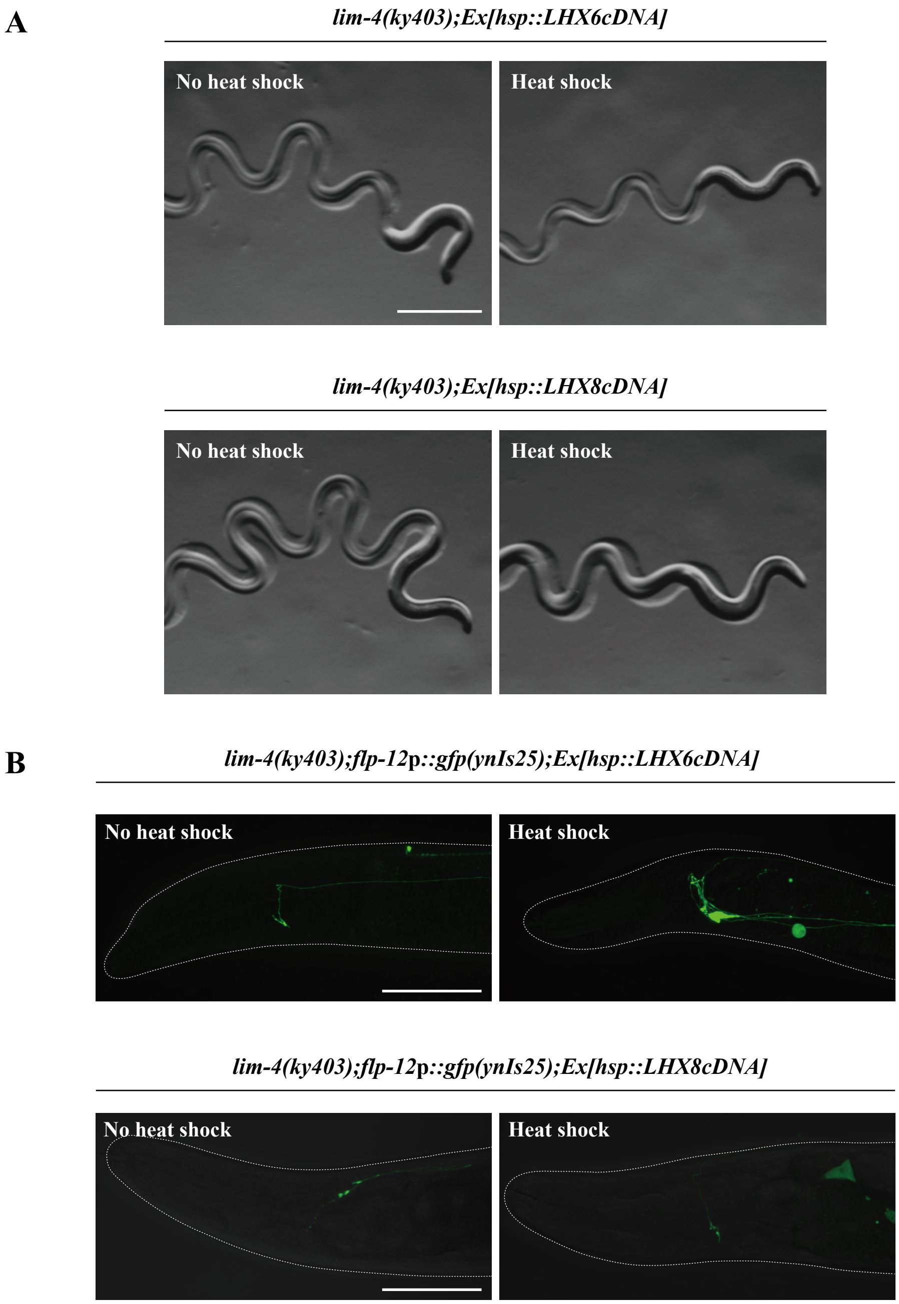

Supplement: S12 Fig — Images are derived from a light microscopy image (A: Scale bar: 0.5 mm) and from z-stacks of confocal microscopy images (B: Scale bar: 50 μm). (PDF) [file pgen.1005480.s017.pdf]
